# Hierarchical Nanostructures as Acoustically Manipulatable Multifunctional Agents in Dynamic Fluid Flow

**DOI:** 10.1002/adma.202404514

**Published:** 2024-10-14

**Authors:** Dong Wook Kim, Paul Wrede, Hector Estrada, Erdost Yildiz, Jelena Lazovic, Aarushi Bhargava, Daniel Razansky, Metin Sitti

**Affiliations:** ^1^ Physical Intelligence Department Max Planck Institute for Intelligent Systems 70569 Stuttgart Germany; ^2^ Institute of Pharmacology and Toxicology and Institute for Biomedical Engineering University of Zürich Zürich 8057 Switzerland; ^3^ Institute for Biomedical Engineering ETH Zürich Zürich 8093 Switzerland; ^4^ School of Medicine and College of Engineering Koç University Istanbul 34450 Turkey

**Keywords:** acoustic manipulation, dynamic fluid, hierarchical nanostructures, medical imaging, microrobots

## Abstract

Acoustic waves provide a biocompatible and deep‐tissue‐penetrating tool suitable for contactless manipulation in in vivo environments. Despite the prevalence of dynamic fluids within the body, previous studies have primarily focused on static fluids, and manipulatable agents in dynamic fluids are limited to gaseous core‐shell particles. However, these gas‐filled particles face challenges in fast‐flow manipulation, complex setups, design versatility, and practical medical imaging, underscoring the need for effective alternatives. In this study, flower‐like hierarchical nanostructures (HNS) into microparticles (MPs) are incorporated, and demonstrated that various materials fabricated as HNS‐MPs exhibit effective and reproducible acoustic trapping within high‐velocity fluid flows. Through simulations, it is validated that the HNS‐MPs are drawn to the focal point by acoustic streaming and form a trap through secondary acoustic streaming at the tips of the nanosheets comprising the HNS‐MPs. Furthermore, the wide range of materials and modification options for HNS, combined with their high surface area and biocompatibility, enable them to serve as acoustically manipulatable multimodal imaging contrast agents and microrobots. They can perform intravascular multi‐trap maneuvering with real‐time imaging, purification of wastewater flow, and highly‐loaded drug delivery. Given the diverse HNS materials developed to date, this study extends their applications to acoustofluidic and biomedical fields.

## Graphical abstract

Flower‐like hierarchical nanostructure microparticles can be trapped by focused ultrasound and manipulated within high‐velocity dynamic fluid flow. The wide range of design versatility, substantial surface area, and biocompatibility enable such microparticles to function as acoustically manipulatable medical imaging contrast agents and microrobots.

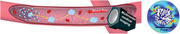

## 1 Introduction

Acoustic manipulation utilizes sound waves to control small objects in liquids or gases.^[^ ^1^, ^2^, ^3^, ^4^ ^]^ In comparison to other contactless manipulation techniques,^[^ ^5^, ^6^, ^7^, ^8^ ^]^ acoustic manipulation not only allows precise adjustments of frequency and intensity but also provides non‐invasive yet highly effective tissue‐penetrating capabilities.^[^ ^1^, ^2^ ^]^ Additionally, acoustic manipulation can be implemented in a highly biocompatible manner without introducing materials with unaccustomed magnetic or dielectric properties.^[^ ^5^ ^]^ Consequently, this approach has been extensively explored across various fields, including biomedical engineering, materials science, and physics.^[^ ^2^ ^]^ Previous studies have employed standing or traveling waves to propel, levitate, or collect nano‐ and micro‐scale objects in fluidic environments.^[^ ^1^, ^2^, ^3^, ^4^ ^]^ However, the majority of these studies have mainly taken place in static fluids, thus constraining their applicability in biological environments where dynamic fluids are more prevalent.

Acoustic manipulation within dynamic fluids has been accomplished using only a limited number of microbubbles^[^ ^9^, ^10^, ^11^, ^12^, ^13^ ^]^ or gas vesicles‐expressed bacteria.^[^ ^14^ ^]^ Their rigid shells and elastic gaseous cores induce strong oscillation, enabling acoustic trapping at flow velocities of up to 10 or 26 mm s^−1^.^[^ ^9^, ^13^ ^]^ However, achieving acoustic trapping of these microbubbles required either waveform modulation^[^ ^9^, ^14^ ^]^ or multiple waves from orthogonally‐positioned transducers.^[^ ^10^, ^11^ ^]^ In waveform modulation, the phase, frequency, or amplitude of the acoustic wave was altered, such as by modulating the wave into a tornado‐like vortex form.^[^ ^9^ ^]^ In the case of orthogonally‐positioned transducers, a standing wave was created by using either 2 or 4 opposing transducers or a reflective surface.^[^ ^10^, ^11^ ^]^ Due to their complexity, both approaches pose challenges in inhomogeneous environments like the living body. The necessity for acoustic wave modification and limited performance under higher (realistic) flow rates present challenges for real‐time 3D monitoring with medical imaging.^[^ ^5^ ^]^ Consequently, in vivo manipulation of microbubbles has been confined to superficial tissues, such as dorsal skin vessels in mice,^[^ ^9^, ^14^ ^]^ zebrafish embryo vasculature,^[^ ^11^ ^]^ or surgically opened brain vasculature,^[^ ^13^ ^]^ where direct optical or fluorescence microscopy observations were feasible. Fragile gaseous cores and reliance on specific shell materials, such as lipids or proteins,^[^ ^15^ ^]^ limit microbubble design and use. It has been reported that microbubbles rupture if the ultrasound mechanical index (MI) exceeds 1.0,^[^ ^16^ ^]^ and their gas cores and shell layers are sensitive to temperature and pH changes,^[^ ^15^ ^]^ restricting further modifications. Given these limitations, there is a need to explore and develop a novel category of materials that are acoustically maneuverable in realistic dynamic fluid environments, like the blood circulatory system, while offering more material design options.

It is widely understood that structural alternations to an object lead to changes in its response to surrounding mechanical waves. This phenomenon has not only driven organisms to evolve and create unique hierarchical structures within their bodies,^[^ ^17^, ^18^ ^]^ but also has influenced the designs of modern architecture and products. This insight leads us to investigate hierarchical nanostructures (HNS) as potential material structures for acoustic manipulation. Over the decades, numerous materials of HNS mimicking the structures of living organisms have been developed, benefiting from their facile self‐assembly property in solution‐based synthesis.^[^ ^19^, ^20^, ^21^, ^22^, ^23^, ^24^ ^]^ HNS feature complex yet reproducible architectures, constructed from low‐dimensional nanoscale building blocks, with their performance optimized through the coupling of these block properties.^[^ ^20^ ^]^ So far, scientists have mainly focused on maximizing the surface area of HNS to enhance reactivity, loading capacity, and sensitivity, and it has led to the discovery of widespread applications in energy storage,^[^ ^20^ ^]^ catalysis,^[^ ^21^, ^22^ ^]^ water purification,^[^ ^23^ ^]^ and sensing.^[^ ^24^ ^]^

**Figure 1:**
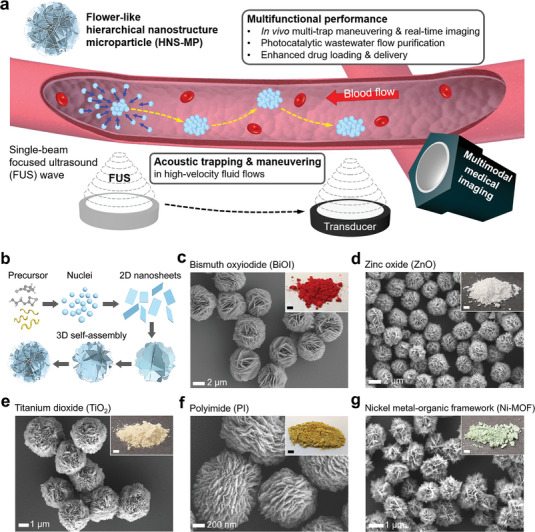
Acoustically maneuverable flower‐like hierarchical nanostructure microparticles (HNS‐MPs) in dynamic fluid flow. a) Conceptual illustration of HNS‐MPs that are acoustically trapped and maneuvered in high‐velocity fluid flow and their application as microrobots and multimodal medical imaging contrast agents. b) Schematic hydrothermal synthesis process of HNS‐MPs. c−g) Scanning electron microscopy (SEM) images of the 5 different HNS‐MPs. Inset photographs display each mass‐produced MPs (scale bar: 5 mm).

Herein, to meet the demand for a novel type of acoustically maneuverable materials suitable for dynamic fluid environments, we propose flower‐like HNS‐microparticles (HNS‐MPs),^[^ ^20^ ^]^ which are composed of hierarchically assembled 2D nanosheets. This nanosheets‐assembled structure enables HNS‐MPs to be acoustically trapped by a focused ultrasound (FUS) wave and manipulated within the high‐velocity fluid flow (up to 60 mm s^−1^). This achievable flow velocity corresponds to blood flow within veins or arterioles and exceeds that of previously reported gas‐filled particles. **Table** **1** compares our HNS‐MPs with the previously developed gas‐filled particles used in in vivo fluidic environments. Along with exceptional acoustic maneuverability, HNS‐MPs offer a substantially high surface area and a wide range of options for particle materials and property modifications. This enables them to be utilized as acoustically manipulatable medical imaging contrast agents and microrobots. Specifically, we demonstrated in vivo multi‐focal HNS‐MPs trapping and real‐time medical imaging within mice veins, as well as microrobot‐driven photocatalytic wastewater purification and drug delivery (**Figure** **1**a).

**Table 1:** Comparison of previously reported acoustically manipulatable agents with our work.

Trapping agent	Maximum fluid flow velocity	Acoustic wave properties	Acoustic trapping and manipulation environments	In vivo imaging method	Refs.
Flower‐like hierarchical nanostructure microparticles (HNS‐MPs)	59.5 mm s^−1^	‐ Single‐beam transducer (Traveling wave, 2 MHz, 0.2−0.25 V_pp_) ‐ Transducer array (512 elements, 3 MHz)	‐ Tubes (Diameter: 0.5, 3.18, 4.77 mm) ‐ Microfluidic setup ‐ Mouse femoral vein (in vivo)	3D optoacoustic tomography imaging	Our work
Microbubbles	26 mm s^−1^	‐ Tornado‐inspired acoustic vortex tweezer ‐ Traveling wave, 3 MHz	‐ Microfluidic setup ‐ Mouse dorsal skin vessels using a skin‐fold window chamber (in vivo)	Fluorescent microscopy imaging	[9]
Microbubbles	Blood flow of zebrafish embryo vasculature (Unknown)	‐ Four orthogonally positioned transducers ‐ 4.0−4.25 MHz, 10−17.5 V_pp_	‐ Zebrafish vasculature (in vivo) (Zebrafish embryo is positioned in a microchannel chamber surrounded by 4 transducers)	Inverted optical microscopy	[11]
Microbubbles	10 mm s^−1^	‐ Transducer array, 18 elements ‐ 490 kHz, 35−44 V_pp_	‐ Mouse brain vasculature opened by cranial window surgery (in vivo)	Fluorescent microscopy imaging	[13]
Gas vesicles‐expressed bacteria	Blood flow of mouse dorsal skin vessels (Unknown)	‐ Transducer array, 64 elements (8 × 8), 3 MHz	‐ Polydimethylsiloxane cavity ‐ Mouse dorsal skin vessels using a skin‐fold window chamber (in vivo)	Fluorescent microscopy imaging	[14]

## 2 Results and Discussion

### 2.1 Acoustic Trapping of HNS‐MPs in Dynamic Fluid Flow

To test the effectiveness and reproducibility of 2D nanosheet‐assembled flower‐like HNS‐MPs in acoustic trapping across different materials, we synthesized 3 inorganic MPs (bismuth oxyiodide (BiOI), zinc oxide (ZnO), titanium dioxide (TiO_2_)), one polymeric MP (polyimide (PI)), and one inorganic‐organic hybrid MP (nickel metal‐organic framework (Ni‐MOF)), and evaluated them for FUS‐driven acoustic trapping under flow conditions. These HNS‐MPs were mass‐synthesized using a commonly employed hydrothermal process.^[^ ^20^ ^−^ ^22^ ^]^ This process involves nucleation of precursor ions or molecules, oriented growth of nuclei into 2D nanosheets, and continuous nanosheets self‐assembly into complex, flower‐like HNS‐MPs (Figure 1b). Each synthesized HNS‐MP exhibits uniform morphology and size, with average particle diameters of 4.9 µm (BiOI), 3.3 µm (ZnO), 3.3 µm (TiO_2_), 1.1 µm (PI), and 1.6 µm (Ni‐MOF) (Figure 1c–g; Figures S1–S3, Supporting Information).

**Figure 2:**
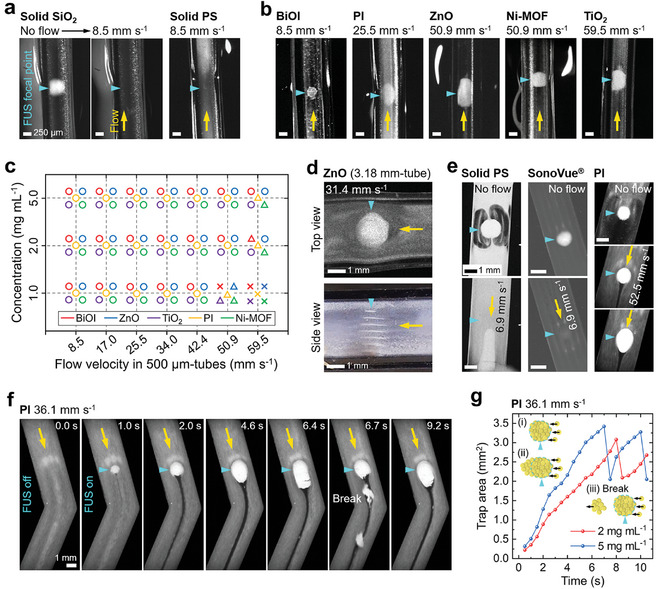
Observation of acoustic trapping under dynamic fluid flow. a, b) Top‐view of the solid SiO_2_ and PS MPs (a), and 5 HNS‐MPs (b) flowing inside the 500 µm‐diameter tubes while focused ultrasound (FUS) was activated. The cyan triangle marks the focal point of FUS and the yellow arrow indicates the direction of water flow. c) Summary of acoustic trapping performance of different HNS‐MPs at various concentrations and flow velocities. The performance test was conducted by using the 500 µm‐diameter tubes shown in Figure 2b. “°” indicates successful trapping in established flow, “△” corresponds to trapping only when a trap is formed before flow, and “×” implies no trapping. d) Top‐ and side‐views of the ZnO MPs trapped inside the 3.18 mm‐diameter tube under water flow. e) Photographs showing the trap formation of solid PS MPs, SonoVue microbubbles, and PI MPs under static flow conditions, and their changes with water flow. f) Time‐dependent photographs showing the formation of a PI MPs trap under an established water flow and its changes inside the microfluidic channel. g) Changes in the area of PI MPs trap over time with different concentrations. The FUS was activated at 0 s.

These HNS‐MPs were compared with solid and homogenous 2 µm‐diameter silica (SiO_2_) and 3 µm‐diameter polystyrene (PS) MPs in terms of their acoustic trapping and manipulation performance under various fluid flow velocities (**Figures** **2**a,b, Figures S4 and S5, and Movie S1, Supporting Information). First, they were injected into the 500 µm‐inner diameter tubes in static or dynamic water flow and subjected to a 2 MHz FUS of 0.42 MPa. The distance between the tube and the transducer was 85 mm, and the space between them was filled with water. The position of the transducer (i.e., the position of the FUS focal point) was adjusted using a three‐axis motor. In static flow, SiO_2_, PS, and HNS‐MPs were trapped at the FUS focal point. However, when the flow commenced (8.5 mm s^−1^), the trapped solid SiO_2_ and PS MPs flowed away, and no new traps were formed. Also, it was not possible to trap these solid MPs in the middle of an established water flow.

In contrast, under water flow with velocities ranging from 8.5 to 59.5 mm s^−1^, the 5 HNS‐MPs not only maintained their traps formed under static flow but also formed traps in the middle of the already established flow. These traps moved along with or against the flow as the FUS focal point shifted. The flow velocities at which HNS‐MPs can be trapped, with a maximum of 60 mm s^−1^, correspond to the blood flow in veins or small arterioles.^[^ ^25^ ^]^ Figure 2c summarizes trapping for the 5 HNS‐MPs inside the 500 µm‐tubes at various concentrations and flow velocities. “°” indicates successful trapping in established flow, “△” indicates trapping only when a trap is formed before the flow, and “×” indicates no trapping. BiOI, PI, and Ni‐MOF MPs showed reduced trapping efficiency at a water flow velocity of 59.5 mm s^−1^. Other MPs, at concentrations of 2.0 and 5.0 mg mL^−1^, were consistently trapped up to 59.5 mm s^−1^, but their trapping efficiency decreased at 1.0 mg mL^−1^. BiOI MPs exhibited a greater tendency to adhere to the surfaces of the tubes compared to the other MPs, likely leading to lower concentration than the intended one and disrupting the trapping process. The reduction in trapping efficiency for smaller MPs (PI and Ni‐MOF) and at lower concentrations will be discussed in the following section.

The HNS‐MPs in the 500 µm‐diameter tubes create a single trap with a 2D shape (Figure 2c; Movie S2, Supporting Information). In larger tubes with inner diameters of 3.18 or 4.77 mm, ZnO and TiO_2_ MPs formed multiple 2D traps vertically at regular intervals within the water flow at various flow velocities (Figure 2d; Figure S6, and Movie S3, Supporting Information). The area of the circular ZnO traps in the 3.18 mm‐diameter tube was ≈2.5 mm^2^ (i.e., the radius of 0.9 mm), and the distances between the vertically formed multiple traps were ≈400 µm. This explains the formation of a non‐circular single MPs trap within the 500 µm‐diameter tubes, where the space is too narrow to form multiple full‐sized traps. Additionally, we trapped BiOI MPs in porcine blood flow and confirmed similar trapping performance as in water flow (Figure S7, Supporting Information).

We further tested the acoustic trapping of solid MPs, SonoVue microbubbles, and HNS‐MPs within a microfluidic channel, which has a width of 3 mm and a height of 0.4 mm (Figure 2e). For a rectangular channel with a high aspect ratio (width to height), the flow velocity near the center is higher compared to the edges.^[^ ^26^ ^]^ This can lead to more complex flow distributions and higher velocity gradients compared to cylindrical tubes. When the FUS was driven under static flow, solid PS, SonoVue microbubbles and PI HNS‐MPs formed a single trap at the FUS focal point. However, as soon as the water flow (velocity of 6.9 mm s^−1^) commenced, the trapped PS MPs and microbubbles flowed away or dispersed into the water. In contrast, the PI MPs trapped under static conditions maintained their trap against a flow velocity of 52.5 mm s^−1^.

The HNS‐MPs also formed a trap in the middle of flowing water, and the trap was manipulated within the microfluidic channel by adjusting the FUS focal point position (Figure 2f; Movie S4, Supporting Information). Figure 2f,g shows the moment of PI MPs trapping and the time‐dependent changes of the PI MPs trap under a water flow velocity of 36.1 mm s^−1^. Upon FUS activation, PI MPs were immediately trapped, forming a clear circular shape within 5 s. As new MPs entered the front of the trap, previously trapped MPs moved toward the rear, elongating the trap from an airfoil to a bullet shape with a tail (at 6.4 s). These shapes are widely known hydrodynamic profiles reducing drag force in dynamic fluids.^[^ ^26^ ^]^ However, the tails soon broke off and were flushed away, causing the trap to revert to its circular shape (at 6.7 s). This repeated breakage and elongation caused fluctuations in the trap area, but the initial circular trap, with an area of ≈2.5 mm^2^, was maintained over time. Along with PI MPs, we demonstrated the trapping and positional manipulation of ZnO MPs in the channels under water flow velocities of up to 55.6 mm s^−1^ (Movie S5, Supporting Information).

The potential impact of air trapped between nanosheets in HNS‐MPs on acoustic trapping performance was addressed with experiments in degassed‐ and non‐degassed water. A comparative overview (Figure S8, Supporting Information) reveals no prominent difference in trapping performance among BiOI, ZnO, and PI MPs. Therefore, the potential presence of trapped air between the nanosheets is neglectable for the effectiveness of acoustic trapping.

### 2.2 Simulation and Physical Insights into HNS‐MPs Acoustic Trapping

**Figure 3:**
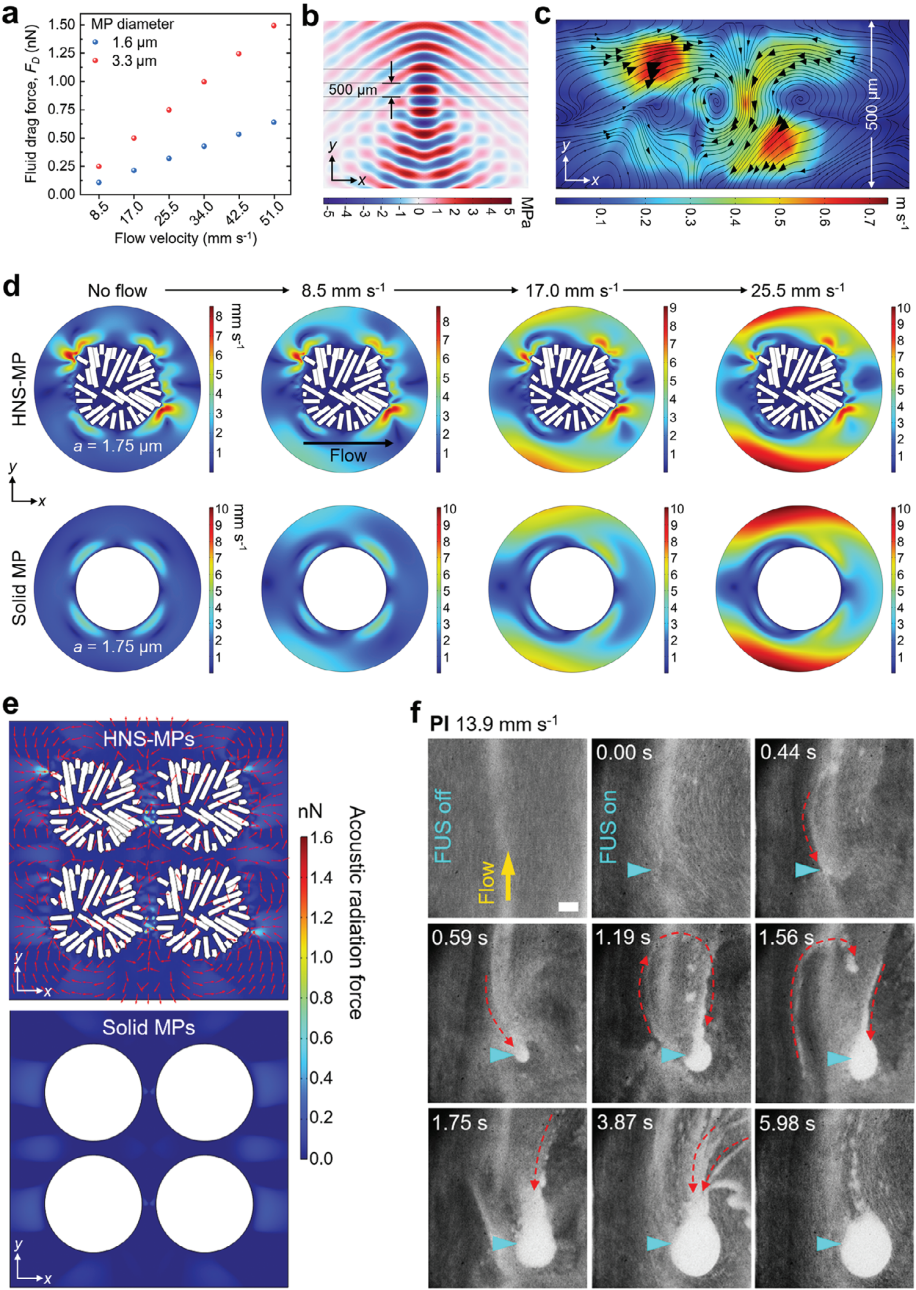
Physical insights into acoustic trapping mechanism of HNS‐MPs. a) Numerical simulation of the flow drag force acting on different‐sized MPs at various flow velocities. b) Simulated pressure field inside and ≈500 µm inner‐diameter tube caused by the 2 MHz single‐lens FUS transducer. c) Acoustic streaming pattern simulated inside the 500 µm inner‐diameter tube caused by the 2 MHz FUS transducer. The tubing is filled with water and assumed to be inviscid. d) Secondary acoustic streaming around HNS‐MP (top) and solid MP (bottom) under static conditions and with different fluid flows applied from left to right. Both particles, with a radius (*a*) of 1.75 µm are positioned at the center of the FUS focal point. e) Inter‐particle acoustic radiation force between multiple MPs. Red arrows indicate the direction of the force. f) High‐speed camera images capturing the motion of PI MPs at the moment of trapping (Scale bar: 1 mm). Red dashed arrows show the flow direction of the MPs.

To understand the physics underlying the trapping mechanism of HNS‐MPs, we conducted finite element simulations (COMSOL 6.2) and numerical simulations to analyze the various acoustic forces acting on the particles. The flow chart and used parameters for our simulations are presented in Figures S9 and S10 (Supporting Information). The trapping condition in fluid flow can be described by a simple force balance: *F_T_* ≥ *F*_*D*_, where *F*_*T*_ is the total trapping force, and *F*_*D*_ is the drag force exerted by the fluid flow. We numerically calculated the *F_D_* for different fluid flow velocities, where we demonstrated successful trapping (**Figure** **3**a). The *F_D_* increased proportionally with flow velocities, and the larger‐diameter HNS‐MPs experienced higher *F*_*D*_in the flow. The amplitude of *F*_*D*_allowed us to estimate the overall required *F_T_*, which mainly includes acoustic radiation force *F_R_*, acoustic streaming‐induced drag force *F_AD_*, as well as secondary acoustic forces, and other inter‐particle forces, such as electrostatic interactions.

The pressure field generated by the 2 MHz transducer in the tubes or channels is the origin of all arising acoustic forces (Figure 3b; Figure S11, Supporting Information). To determine whether the motion of HNS‐MPs is governed by *F_R_* or *F_AD_*, we calculated the dimensionless parameter $\kappa =\frac{2\;\pi r}{\lambda }$, where *r* is the particle radius, and λ is the wavelength of the fluid. According to Skowronek et al.,^[^ ^27^ ^]^ If κ < 1, particle motion within the acoustic pressure field is dominated by *F_AD_*, so that particles with small sizes and at low frequencies follow the motion of the flow field. For all HNS‐MPs with an average diameter in the range between 1.1 and 4.9 µm at 2 MHz FUS, κ falls within the range of 10^−2^ to 10^−3^ (Table S1, Supporting Information), indicating that the motion of HNS‐MPs follows the acoustic streamlines generated by the FUS.

As experimentally demonstrated in Figure 2e and simulated in Figure 3c and Figure S12 (Supporting Information), the FUS‐driven acoustic streaming generates vortices around the high‐pressure region, causing fluid to flow toward it. The simulated velocity of this FUS‐induced streaming flow is higher than that of the applied fluid flow, which ranges from 0 to 59.5 mm s^−1^. This streaming exerts *F_AD_* on the HNS‐MPs, dragging them toward the focal point and increasing the local particle concentration there. While *F_R_* depends on the radius, compressibility, and density of the particles (Experimental Part, Equations 1 and 2), *F_AD_* depends only on the radius of the particles. Therefore, we would expect similar trapping for particles of the same size if *F*_*AD*_ is the main driving force. While this holds true in static conditions, where various particles were stably trapped at the focal point, the situation changes in dynamic conditions. In the presence of flow, solid MPs not only failed to maintain their trap formed under static conditions but also could not form a trap in the middle of the flowing fluid (See Figure 2a,e), This observation indicates that other forces play a critical role.

The multiple 2D nanosheets comprising the HNS‐MPs form sharp, pointed tips on the surface of the particles (See Figures S2 and S3, Supporting Information). It is well established that such sharp structures generate secondary acoustic streaming with high‐velocity amplitudes at the tips.^[^ ^28^ ^−^ ^30^ ^]^ Recently, Harley et al.,^[^ ^30^ ^]^ show that sharp edge tips in 3D sub‐wavelength microstructures create acoustic streaming vortices with significant velocities, with this velocity increasing as the angles of the edges become sharper. As a result, the higher velocity vortices induced a minimum Gor'kov potential at the tips, causing the gradient of the acoustic potential field to exert *F_R_* on the surrounding particles, directing them toward the microstructure.

We conducted simulations of secondary acoustic streaming around HNS‐MPs and solid MPs with a radius of 1.75 µm under fluid flow at various velocities (Figure 3d; Figure S13, Supporting Information). Both types of MPs were positioned at the focal point, with water flow flowing from the left to right of the particle. Unlike the solid MPs, the nanosheet tips on the surface of HNS‐MPs generated secondary acoustic streaming vortices with high‐velocity amplitudes. This local amplification of streaming velocity creates a region of minimum Gor'kov potential (Experimental Part, Equation 5) at the nanosheet tips, attracting nearby HNS‐MPs and resulting in the formation of a clustered trap. Such localized secondary acoustic streaming was not observed in solid particles with smooth surfaces. Due to the complex geometry of the 3D HNS‐MPs, our simulation was conducted in 2D and only depicted a cross‐section of the MP. However, given the sharp, pointed nanoscale tips observed across the entire surface of HNS‐MPs, the reduction in Gor'kov potential around these MPs would exert substantial *F_R_* on the nearby MPs, attracting them to the FUS focal point and forming a trap with a volume much larger than that of a single MP.

Additionally, we calculated the inter‐particle *F*_R_ between the 4 HNS‐MPs, and the 4 solid MPs, as shown in Figure 3e. The inter‐particle *F*_R_ between the HNS‐MPs (with a diameter of 1.75 µm) was greater than 0.6 nN, reaching a maximum of 1.6 nN, while the solid MPs exhibited no inter‐particle forces. The red arrows indicate the direction of the *F*_R_. Notably, the inter‐particle *F*_R_ between the HNS‐MPs surpassed the calculated *F*_D_ acting on the 1.6 µm‐diameter MPs (Figure 3a), strongly supporting the underlying mechanism that allows the clustered HNS‐MPs to maintain their trap under the applied flow.

Therefore, summarizing the mechanism of acoustic trapping of HNS‐MPs, when FUS is applied to the flow of HNS‐MPs, the FUS‐driven high‐velocity *F_AD_* drags the MPs toward the focal point. Meanwhile, secondary acoustic streaming at the nanosheet tips creates a region of minimum Gor'kov potential, which leads to the agglomeration of MPs at the FUS focal point. Since the *F_R_* is proportional to the volume of the particle or particle cluster (Experimental Part, Equation 1), and the volume of the agglomerated MPs trap is much larger than that of a single MP, the *F_R_* acting on the trap overcomes the *F_D_*, allowing for the trap to be stable against the fluid flow.

We also validated the aforementioned mechanism through experimentation. Figure 3f presents high‐speed camera images that capture the motion of PI MPs at the moment of trapping. Immediately upon activating the FUS (0.00 s), the flow of PI MPs transitioned from laminar to turbulent motion due to the combined effects of the applied flow and FUS‐driven acoustic streaming. Since the velocity of the FUS‐driven streaming flow exceeded that of the applied flow, we could clearly observe the dragging and subsequent trapping of PI MPs toward the focal point (indicated by the red dashed arrows), following the streaming pattern. Through continuous dragging and trapping of MPs, the size of the trap continued to increase for ≈5.00 s, and once it reached the size of the focal point, it maintained a stable shape (5.98 s).

Experimental data show that forming a trap under static conditions before applying flow or increasing the concentration of HNS‐MPs in fluids enhances trapping performance (Figure 2c). Trapping first under static conditions forms a large and high packing density MPs trap. Since the secondary acoustic streaming around the HNS‐MPs are very short distances, high packing density is crucial to minimize inter‐particle distances, thus enabling stable trap formation. Also, at higher initial MPs concentrations, the inter‐particle distances at the FUS focal point are reduced, making it easier to attract more MPs to form a trap. Particle diameter affects all discussed forces (*F_D_*, *F_R_*, and *F_AD_*) and the packing density, making it crucial for trapping efficiency. As shown in Figure 2c, the trapping efficiency of smaller diameter PI and NI‐MOF MPs degraded at lower particle concentrations and higher flow velocities.

### 2.3 HNS‐MPs as Contrast Agents in Multimodal Medical Imaging

**Figure 4:**
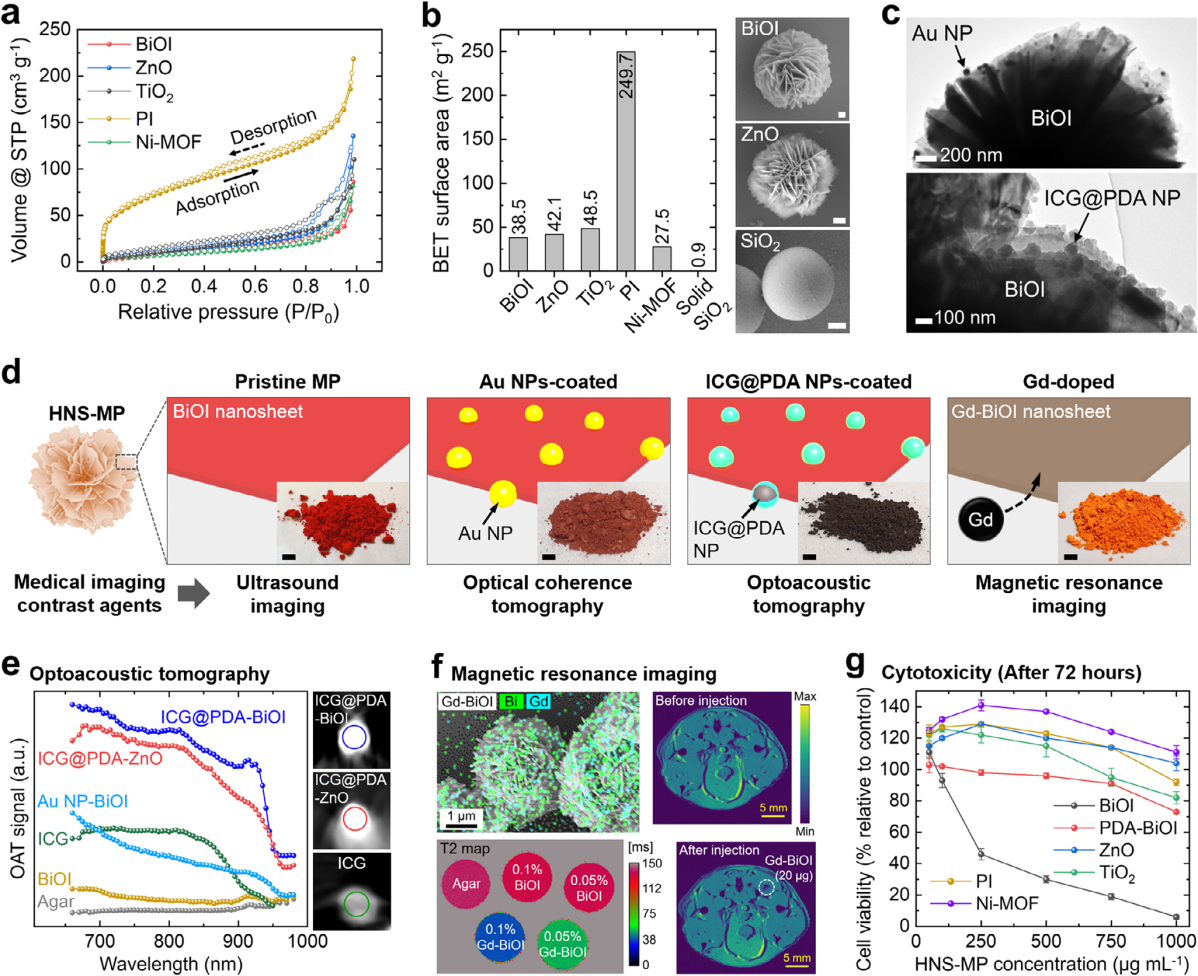
High surface area and biocompatibility of HNS‐MPs for use as multimodal imaging contrast agents. a, b) N_2_ adsorption‐desorption isotherms of the 5 HNS‐MPs measured at standard temperature and pressure (STP) (a) and their calculated Brunauer‐Emmett‐Teller (BET) surface area compared with solid SiO_2_ MP (b). The right SEM images in (b) show a single MP of BiOI, ZnO, and SiO_2_ (scale bar: 500 nm). c) Transmission electron microscopy (TEM) images of BiOI MPs coated with Au NPs (top) and ICG@PDA NPs (bottom). d) Functionalized HNS‐MPs as contrast agents in multimodal imaging. The inset shows the mass‐produced individual MPs (scale bar: 5 mm). e) Multi‐spectral optoacoustic tomography (OAT) spectrum of 6 different samples. Each sample was dispersed in an agar phantom tube. The right images show the OAT signal from 3 different samples. f) Top left: elemental mapping of the Gd‐BiOI MPs. Bottom left: T2 signal mapping of the 5 different samples. Right: T2 magnetic resonance images of ex vivo mice before and after Gd‐BiOI MPs injection. g) Viability of human skin fibroblast cells after 72 h of incubation with various concentrations of different HNS‐MPs.

The unique nanosheet‐assembled structure of HNS‐MPs provides a high surface area, enhancing material loading and reactions with external substances. We leveraged this high surface area to coat functional nanoparticles or dope desired atoms into HNS‐MPs, creating acoustically manipulatable imaging contrast agents and microrobots. The Brunauer‐Emmett‐Teller (BET) surface area, measured from the N_2_ volume adsorbed and desorbed from the particle surface over a range of relative pressures (P/P_0_) were: BiOI (38.5 m^2^ g^−1^), ZnO (42.1 m^2^ g^−1^), TiO_2_ (48.5 m^2^ g^−1^), PI (249.7 m^2^ g^−1^), and Ni‐MOF (27.5 m^2^ g^−1^) (**Figure** **4**a,b). The PI MPs exhibited the highest surface area due to their intrinsic spaces between polymer chains.^[^ ^21^ ^]^ Compared to solid SiO_2_ MPs (2 µm diameter) having a BET surface area of only 0.9 m^2^ g^−1^, the HNS‐MPs had substantially higher surface areas (Figure 4b).

To acoustically manipulate HNS‐MPs in vivo environments without direct observation, medical imaging systems are essential. We utilized HNS‐MPs themselves or selectively modified the MPs according to the method of imaging, including ultrasound (US) imaging, optical coherence tomography (OCT), optoacoustic tomography (OAT), and magnetic resonance imaging (MRI). The modifications involved coating with gold nanoparticles (Au NPs), encapsulating with indocyanine green@polydopamine (ICG@PDA) NPs, or doping with gadolinium (Gd) atoms, (Figure 4c,d; Figure S14, Supporting Information). These modifications can be conducted in bulk, and the color of HNS‐MPs is changed based on the process used (Figure 4d).

The US contrast agent efficacy of HNS‐MPs was evaluated using agar phantom samples with different concentrations of BiOI, solid SiO_2_ MPs, and SonoVue (Figure S15, Supporting Information). Compared to SiO_2_ MPs having a single scattering/reflection interface, the BiOI HNS‐MPs exhibited superior US contrast performance owing to the multiple scattering/reflection interfaces from randomly oriented nanosheets.^[^ ^32^ ^]^ The US contrast signal was similar to SonoVue, which is known for its excellent US contrast performance.^[^ ^33^ ^]^ Functionalization, such as Gd‐doping did not affect the US contrast, with the distribution of Gd‐BiOI MPs clearly visualized in mouse vasculature ex vivo (Movie S6, Supporting Information).

For higher‐resolution imaging with OCT and OAT,^[^ ^5^, ^34^ ^−^ ^36^ ^]^ HNS‐MPs were coated with Au NPs or ICG@PDA NPs, respectively (Figure 4c). Au NP‐BiOI MPs demonstrated a considerable OCT signal at all concentrations, unlike BiOI MPs and SonoVue (Figure S16, Supporting Information). This efficient detection of MPs is attributed to the high density of Au NPs with strong surface plasmon resonance^[^ ^34^ ^]^ decorated on the large surface area of HNS‐MPs. Plasmonic Au NPs also effectively convert the absorbed laser pulse into ultrasound, thus enhancing OAT contrast.^[^ ^34^ ^−^ ^36^ ^]^ Au NP‐BiOI MPs manifested monotonically increasing OAT responses when shifting the wavelength from 950 nm down to 650 nm, unlike pristine BiOI (Figure 4e). Although we successfully visualized injected Au NP‐BiOI MPs in the peritoneal cavity of ex vivo mice, their OAT signal overlapped with hemoglobin and melanin signals (Figure S17, Supporting Information). To achieve high‐contrast imaging, we incorporated ICG into HNS‐MPs with distinct absorption near 800 nm.^[^ ^5^, ^36^ ^]^ Coating BiOI or ZnO HNS‐MPs with PDA NPs, followed by loading ICG through electrostatic and hydrophobic interactions, enabled both ICG@PDA‐BiOI and ‐ZnO MPs to exhibit a distinct OAT signal at 815 nm, with intensities higher than that of the bare ICG solution (Figure 4e). Minimal amounts of HNS MPs were successfully imaged using MRI (Figure 4f). Gd was atomically doped into BiOI during synthesis without altering its hierarchical structure. Gd‐BiOI MPs served as T2‐weighted contrast agents with r_2_/r_1_ value of 32.8, and even a small injection could be detected ex vivo, highlighting the sensitivity and effectiveness of Gd‐doping (Figure S18, Supporting Information).

Cytotoxicity and hemocompatibility of HNS‐MPs were examined for potential biomedical use. Pristine or modified HNS‐MPs were incubated with human skin fibroblast cells to assess cell viability (Figure 4g). BiOI MPs exhibited high cytotoxicity, but PDA coating reduced cytotoxicity significantly. ZnO, TiO_2_, Ni‐MOF, and PI MPs rather promoted cell growth without significant cytotoxicity, and we confirmed the same effect across 2 tests (Figures S19 and S20, Supporting Information). During the initial 24 h of incubation, cell viability decreased with increasing MP concentration. However, from 48 h, moderate MP concentrations (50–500 µg mL^−1^) led to gradual cell growth with viability exceeding 120%. Notably, at 1000 µg mL^−1^, where acoustic trapping tests are conducted, PDA‐BiOI, ZnO, Ni‐MOF, and PI MPs exhibited increased cell viability over 3 days. Previous studies suggested that flower‐like HNS of ZnO,^[^ ^37^ ^]^ CaSiO_3_,^[^ ^38^ ^]^ or TiO_2_^[^ ^39^, ^40^ ^]^ promote cell growth and bone tissue regeneration by enhancing cell adhesion, proliferation, and differentiation. Consequently, the promotion of cell growth by HNS‐MPs is attributed to their hierarchical structures. However, at higher concentrations, the effects on cell growth and material cytotoxicity compete and determine the final viability.

In hemocompatibility tests, no hematotoxicity or cellular damage was observed in all experimental groups (ZnO, PI, ICG@PDA‐BiOI, and ICG@PDA‐ZnO) after an agitated blood incubation period (Figure S21, Supporting Information). While the platelet activation and aggregation were investigated in each experimental group, no significant thrombogenicity was observed in all MPs in both 1.0 and 5.0 mg mL^−1^ concentration (Figure S22, Supporting Information).

### 2.4 Simultaneous Multi‐Focal Trapping and Imaging of HNS‐MPs In Vitro

**Figure 5:**
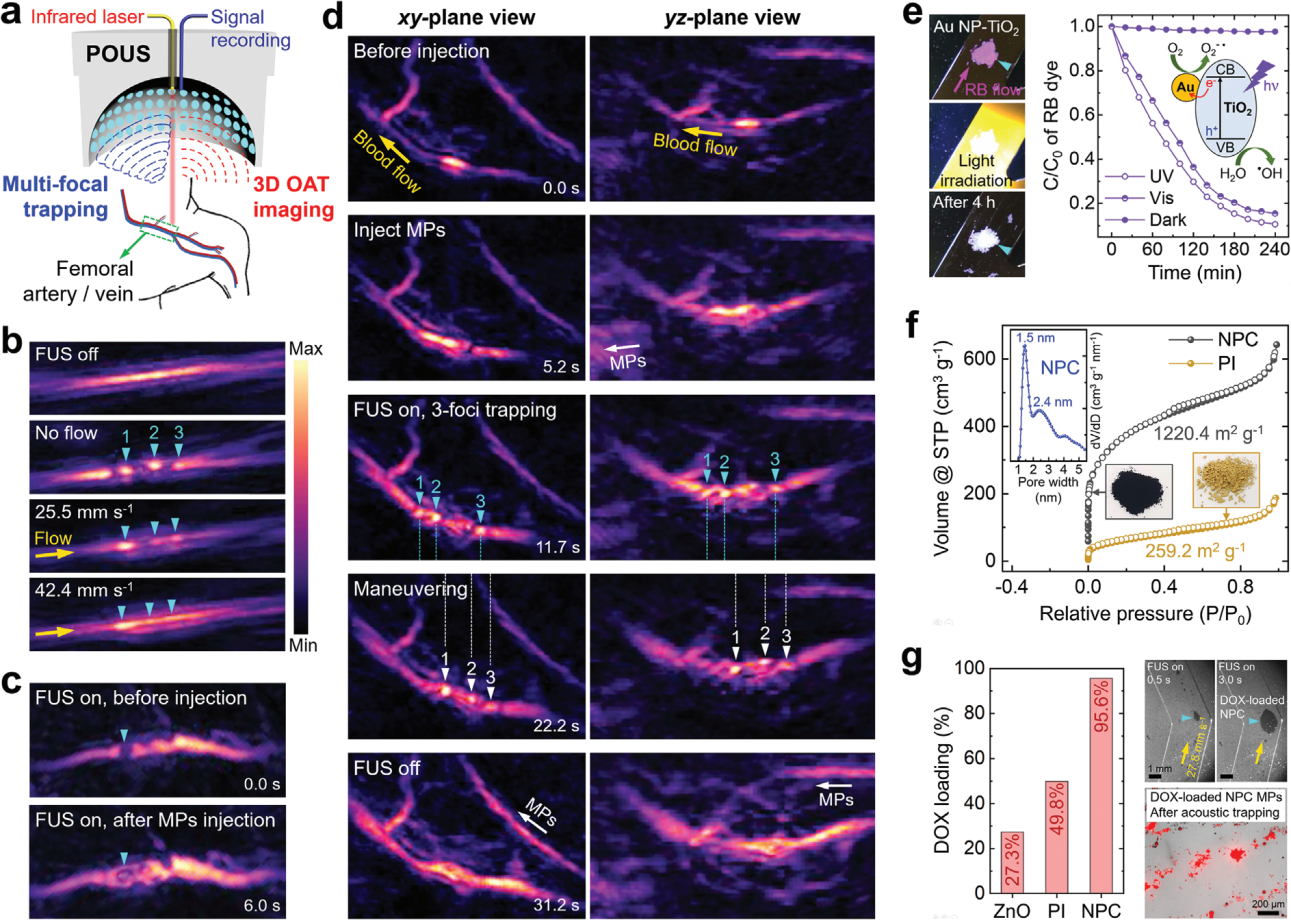
HNS‐MPs as acoustically manipulatable medical imaging contrast agents and multifunctional microrobots. a) High‐resolution parallelized optoacoustic imaging & ultrasound (POUS) system for real‐time acoustic manipulation of HNS‐MPs and tracking with 3D OAT imaging. b) OAT imaging of the triple traps of ICG@PDA‐ZnO MPs within the 500 µm‐diameter tubes under various flow velocities. c) OAT imaging of the femoral vein during FUS activation without and with ICG@PDA‐ZnO MPs inside the vein. d) *xy*‐ and *yz*‐plane OAT imaging views showing the formation of triple ICG@PDA‐ZnO MPs traps within the femoral vein, positional maneuvering, and dissolution of the traps. e) Purification of Rhodamine B (RB) dye‐contained water flow using the Au NP‐TiO_2_ MPs trap under light irradiation and the decrease in relative RB concentration (C/C_0_). The Inset scheme depicts the photocatalytic dye degradation mechanism. f) N_2_ adsorption‐desorption isotherms of polyimide (PI) and nanoporous carbon (NPC) HNS‐MPs with their BET surface area. Pore size distributions of NPC MPs and photographs of PI and NPC MPs are shown in the inset. g) Left: comparison of doxorubicin (DOX) loading efficiency among ZnO, PI, and NPC MPs. Right: Acoustic trapping of DOX‐loaded NPC MPs under water flow, and the fluorescent image of the MPs after trapping experiments. The fluorescent image is overlaid with its corresponding brightfield microscopy image.

In medical contexts, simultaneous manipulation and imaging of drug carriers are crucial for precise targeted navigation. Ideally, such devices should be compact and easily integrated into bedside scenarios. Both US and OAT offer bedside devices and use piezo‐element transducers for signal detection. Therefore, they align well with our proposed FUS trapping technique, which employs the same piezo elements to send and focus acoustic waves at specific positions. Here, we preferred OAT over US since OAT is capable of acquiring an entire 3D volume with a single laser pulse, while conventional US only captures a single plane image at a time (Figure S23 and Movie S6, Supporting Information). For real‐time 3D OAT imaging, we used our home‐built high‐resolution parallelized optoacoustic imaging and ultrasound (POUS) system^[^ ^41^ ^]^ (**Figure** **5**a). It consists of a dense spherical matrix transducer consisting of 512 piezo‐elements. Both the driving voltages and transmission delays can be customized to adjust the precise timing for trapping and imaging. The system allows generating and steering 130 µm‐sized FUS focal spots across a field of view covering ≈1 cm^3^, while the same array is used to capture high‐resolution 3D OAT data in real‐time. This real‐time trapping and imaging are possible because the 24 ms‐long FUS trapping and OAT imaging were rapidly alternated at an effective combined frame rate of 20 volumes per second (vps). Multiple foci can be also created at arbitrary locations, which enables placing acoustic traps into different regions simultaneously and independently from each other. The formation of multiple traps is expected to enhance trapping efficiency, since the first trap slows down the particle flow, making it easier for the last trap to trap particles. This independent multi‐trap manipulation has high potential for targeted drug delivery, as it can trap more therapeutic particles compared to a single trap, potentially increasing therapeutic efficacy.

We first conducted real‐time acoustic trapping and simultaneous OAT imaging of ICG@PDA‐coated HNS‐MPs inside the 500 µm‐diameter tubes (Figure 5b; Figure S24, and Movie S7, Supporting Information). Three foci were arranged consecutively, each operating at a frequency of 3 MHz and a maximum pressure of 2.8 MPa (Figure S25, Supporting Information). In the absence of FUS, we observed a continuous flow of ICG@PDA‐ZnO MPs via OAT imaging at 800 nm wavelength. When FUS was activated in static flow, the MPs were trapped at the focal points, resulting in 3 distinct and clearly visible traps. At flow velocities of 25.5 and 42.4 mm s^−1^, the 3 traps remained visible, although the foremost trap becomes more prominent as it encounters and captures the majority of incoming MPs, whilst the rapid flow of MPs between the 3 traps hinders a clear differentiation between the traps.

To investigate the potential of HNS‐MPs attaching to blood vessels, we conducted real‐time OAT imaging and acoustic trapping of ICG@PDA‐ZnO MPs within tubes and ex vivo blood vessels under the flow of water or porcine blood. We then examined the walls of the tubes and blood vessels (Figure S24, Supporting Information). After trapping and releasing the MP flow, no residual ICG@PDA‐ZnO MPs were found attached to the walls of the tubes or vessels. We observed that only a few MPs flowed into the vessel capillaries and became lodged inside them.

### 2.5 Real‐Time Intravascular Maneuvering and 3D Imaging of HNS‐MPs In Vivo

We accomplished real‐time acoustic multi‐trap manipulation and 3D OAT imaging within the murine femoral vein in vivo, which has a corresponding blood flow velocity of 16 mm s^−1^.^[^ ^42^ ^]^ In contrast to the simple geometry of in vitro tubes, in vivo blood vessels are more complex and difficult to distinguish from their surroundings. Additionally, while particle concentration is constant in in vitro, in vivo conditions cause HNS‐MPs to disperse throughout the vascular system, reducing their local concentration in the blood vessel. Moreover, in vitro experiments involve acoustic waves traveling through water alone, whereas in vivo experiments involve water, ultrasound gel, and tissue. Despite these challenges, our POUS system, designed for in vivo use, offers spatial 3D OAT imaging and precise tissue‐penetrating FUS application.^[^ ^41^ ^]^

For the in vivo test, the mouse was positioned on a moving stage with the POUS system targeting its hindlimb (Figure S26, Supporting Information). The mechanical index (MI) for our POUS system was calculated to be 1.6. It is therefore below the FDA‐approved safety limit of MI = 1.9 for diagnostic imaging.^[^ ^43^ ^]^ Before injecting ICG@PDA‐ZnO MPs (1.0 mg mL^−1^) via the mouse tail vein, we distinguished the target femoral veins with adjacent arteries by alternating the OAT imaging wavelength between 760 and 850 nm (Figure S27, Supporting Information). The distinct absorption characteristics between deoxyhemoglobin and oxyhemoglobin allowed us to confidently differentiate between the arteries and veins. We applied FUS to the femoral vein without MPs (Figure 5c; Movie S8, Supporting Information), resulting in clear identification of red blood cells (RBCs) displaced from the focal point, consistent with previous reports.^[^ ^44^ ^]^ We additionally confirmed this FUS‐driven displacement of RBCs in brain blood vessels (Figure S25, Supporting Information). It has been reported that blood plasma, which makes up half of the blood alongside RBCs, continues to flow regardless of the FUS.^[^ ^45^ ^]^ After injecting ICG@PDA‐ZnO MPs, we observed the formation of a trap at the focal point upon FUS activation.

Next, we conducted real‐time acoustic manipulation and 3D OAT imaging of the triple traps within the femoral vein (Figure 5d; Figure S28, and Movie S9, Supporting Information). Upon injecting ICG@PDA‐ZnO MPs into the tail vein (time point 2), a significant enhancement in the OAT signal intensity occurred, enabling clear visualization of MP flow into the femoral vein. Upon FUS activation (time point 3), 3 distinct MP traps were visualized at each designated focal point. Through controlled stage movement, the positions of the focal points were steered within the vessel, allowing us to track the maneuvering of triple traps along the vein vessel (time point 4). The traps dissolved when FUS was deactivated, leading to the flow of stagnant MPs behind the traps thus causing an increase in the OAT signal within the monitored vessel and its connected surrounding vessels (time point 5). In the future, the physical steering of the FUS focal point could be improved by using a surgical robotic arm^[^ ^46^ ^]^ or electronically controlled beam‐steering techniques,^[^ ^14^ ^]^ this advancement would enable more precise manipulation of acoustic traps over a larger area in complex in vivo environments.

### 2.6 HNS‐MPs as Acoustically‐Driven Multifunctional Microrobots

Various HNS‐MPs offer versatile microrobot functionality^[^ ^47^ ^]^ owing to their unique photocatalytic properties^[^ ^20^ ^−^ ^23^ ^]^ or nanoporosity.^[^ ^21^, ^22^ ^]^ We first employed HNS‐MPs to purify waterflow containing Rhodamine B (RB) dye (Figure 5e). A microfluidic channel containing trapped Au NP‐TiO_2_ MPs was subjected to a circulating flow (27.8 mm s^−1^) of RB dye solution, then the trap was irradiated with ultraviolet or visible green light‐emitting diodes (LEDs). Since the dye solution with MPs flows continuously and the LED‐illuminated area in the microfluidic channel is confined, aligning the Au NP‐TiO_2_ MPs trap with this illuminated area enhances photocatalytic dye degradation. Over 4 h of light irradiation, the dye color faded and the RB absorption peak decreased. This reduction did not occur in the dark or with FUS alone without MPs and light. Degradation of RB dye by MPs flow was minimal when exposed only to light without targeted illumination on the trap (Figure S29, Supporting Information). The Au NPs form the Schottky junction at the interface with semiconducting TiO_2_ to promote the production of reactive oxidants.^[^ ^48^ ^]^ A more detailed dye degradation mechanism is provided in Figure S30 (Supporting Information).

To significantly enhance the cargo loading efficiency in cargo transport, we transformed PI MPs into nanoporous carbon (NPC) MPs. Through pyrolysis/activation of PI,^[^ ^21^ ^]^ the surface area remarkably escalated from 259.2 m^2^ g^−1^ for PI to 1220.4 m^2^ g^−1^ for NPC (Figure 5f). The NPC MPs exhibited a pore size distribution ranging from 1.5 to 2.4 nm. We focused on loading doxorubicin (DOX), a chemotherapeutic agent effective against various cancer types,^[^ ^49^ ^]^ into ZnO, PI, and NPC MPs, allowing for a direct comparison of loading efficiencies. The absorption intensity of DOX was compared before and after agitation with each type of MP, resulting in loading efficiency of 27.3%, 49.8%, and 95.6% for ZnO, PI, and NPC MPs, respectively (Figure 5g; Figure S31, Supporting Information). This substantial enhancement in loading capacity with NPC MPs is attributed to their ultrahigh surface area and nanoscale pores, providing an ideal environment for the absorption of DOX molecules (molecular size < 1.5 nm^[^ ^49^ ^]^). The presence of DOX within a single NPC MP was visually confirmed through fluorescent imaging. Moreover, even after subjecting the DOX‐loaded NPC MPs to a trapping test, the fluorescence intensity remained constant, emphasizing the stability of DOX loading into NPC MPs. We conducted simultaneous OAT imaging and trapping of DOX‐loaded NPC MPs (Figure S32, Supporting Information). NPC MPs were decorated with ICG@PDA NPs and loaded with DOX. At a flow velocity of 50.9 mm s^−1^ within 500 µm‐diameter tubes, we observed the trapping of DOX‐loaded NPC MPs via OAT imaging.

## 3 Conclusion

In this paper, we present flower‐like HNS‐MPs made from 2D nanosheets as a novel acoustically manipulatable material structure in dynamic fluids. These MPs can be trapped by FUS and maneuvered in fluid flows at venous‐ or arteriole‐level velocities (up to 60 mm s^−1^), while serving as imaging contrast agents and microrobots. Through systematic experiments and simulations, we confirmed the consistent efficacy and reproducibility of acoustic trapping across 5 types of flower‐like HNS‐MPs and their variants. This capability is attributed to their hierarchically assembled nanosheets, which influence acoustic streaming and modify the forces acting on the MPs. HNS‐MPs also offer diverse material options, including various semiconducting oxides and polymers, and typically feature high surface areas that enhance material modification. Additionally, HNS‐MPs exhibit low cytotoxicity and high hemocompatibility, making them suitable for biological environments. These advantages enable HNS‐MPs to serve as acoustically manipulatable medical imaging contrast agents and microrobots capable of specific tasks, such as in vivo maneuvering with real‐time 3D OAT imaging, photocatalytic wastewater purification, and highly loaded drug delivery.

Future research could explore HNS‐MPs for advanced biomedical and acoustofluidic applications, such as treatment, diagnosis, microrobotics, and microfluidics, especially in fast‐flowing fluids. While concerns about long‐term effects on metabolism and circulatory exist, biodegradable and biocompatible HNS‐MPs made from hydroxyapatite or calcium phosphate could address these issues.^[^ ^19^ ^]^ We confirmed the degradability, high cell viability, and acoustic trapping capability of hydroxyapatite HNS‐MPs (Figure S33, Supporting Information). Additionally, deploying HNS‐MPs via the catheter and performing FUS‐driven procedures in targeted areas could help minimize side effects.

Lastly, while the present study provides insights into the trapping performance of HNS‐MPs based on experimental data and theoretical models, the exact acoustic and hydrodynamic responses of HNS‐MPs and numerical modeling−considering various particle materials, nanosheet configurations, clustered particles, and channel geometries−remain complex. Further exploration of these elements will be essential to fully elucidate the trapping behavior in dynamic acoustofludic environments.

## 4 Experimental Section

### Synthesis of Bismuth Oxyiodide (BiOI) MPs

First, 0.728 g of bismuth (III) nitrate pentahydrate (Bi(NO_3_)_3_∙5H_2_O, Sigma‐Aldrich) was dissolved into 20 mL ethanol, and 0.249 g of potassium iodide (KI, Sigma‐Aldrich) was dissolved into 40 mL deionized water separately. Then, the KI solution was added drop‐wise into the Bi(NO_3_)_3_∙5H_2_O solution under continuous stirring. The pH value of the mixture was adjusted to 7.0 by adding NH_3_∙H_2_O. Finally, the whole solution was stirred for 3 h at 80 °C. After cooling down, the red precipitate was collected by centrifugation, washed several times with deionized water, and finally dried overnight at 80 °C.

### Synthesis of Zinc Oxide (ZnO) MPs

0.892 g of zinc nitrate hexahydrate (Zn(NO_3_)_2_∙6H_2_O, Sigma‐Aldrich) and 2.118 g of sodium citrate tribasic dihydrate (HOC(COONa)(CH_2_COONa)_2_·2H_2_O, Sigma‐Aldrich) were dissolved into 60 mL deionized water under continuous stirring to form a transparent solution. Subsequently, 0.6 g of sodium hydroxide (NaOH, Sigma‐Aldrich) was added to the solution. After stirring for 2 h at room temperature, the white precipitate was collected by centrifugation, washed several times with deionized water, and finally dried overnight at 80 °C.

### Synthesis of Titanium Dioxide (TiO_2_) MPs

8 mL of titanium (IV) butoxide (Sigma‐Aldrich) was mixed with 40 mL of chloroform (anhydrous, Sigma‐Aldrich), flowed by the addition of 4 mL hydrochloric acid (HCl, Carl Roth, 37 wt.%) dropwise. Then, the whole mixture was transferred into a Teflon‐lined autoclave and maintained at 180 °C for 5 h. After being cooled to room temperature, the white precipitate was collected by centrifugation, washed several times with deionized water, and finally dried overnight at 80 °C.

### Synthesis of Polyimide (PI) MPs

1.78 g of benzidine (Sigma‐Aldrich) as the diamine monomer was added into 60 mL of *N,N*‐dimethylformamide (DMF, anhydrous, Sigma‐Aldrich) under stirring. After the benzidine was completely dissolved, 3.11 g of 3,3′,4,4′‐benzophenonetetracarboxylic dianhydride (BTDA, Sigma‐Aldrich) was added into the above mixture and continuously stirred at room temperature for 10 h for pre‐polymerization. After 10 h, the dark brownish viscous poly(amic acid) (PAA) solution was obtained. 30 mL of PAA solution was transferred into a Teflon‐lined autoclave and maintained at 180 °C for 10 h. After being cooled to room temperature, the yellow precipitate was filtered by filter paper (0.1 µm, regenerated cellulose, Cytiva) and washed with DMF and ethanol several times. The resultant powder was collected after drying overnight at 80 °C. To synthesize the low‐density PI MPs, the DMF was replaced with *N*‐methyl‐2‐pyrrolidone (NMP).

### Synthesis of Nickel Metal‐Organic Framework (Ni‐MOF) MPs

A mixture solution including NiCl_2_∙6H_2_O (0.1785 g, Sigma‐Aldrich), terephthalic acid (0.1245 g, Sigma‐Aldrich), deionized water (4 mL), and DMF (30 mL, anhydrous, Sigma‐Aldrich) was transferred into a Teflon‐lined autoclave and maintained at 140 °C for 10 h. After being cooled to room temperature, the greenish precipitate was collected by centrifugation, washed several times with deionized water, and finally dried overnight at 80 °C.

### Synthesis of Gold Nanoparticles (Au NP)‐Coated HNS‐MPs

Au NP‐BiOI (or TiO_2_) MPs were synthesized through a one‐step chemical reduction of Au (III). 5 mL of aqueous solution of pristine BiOI (or TiO_2_) MPs (10 mg mL^−1^) was prepared, and 50 µL of aqueous HAuCl_4_∙3H_2_O solution (200 mM, Sigma‐Aldrich) was added and stirred for 10 min. After that, 4 mL of aqueous NaBH_4_ (5 mM, Sigma‐Aldrich) was added dropwise and the reaction was continued for 30 min by magnetic stirring. Finally, the dark red (light purple for TiO_2_) precipitate of Au NP‐BiOI (or TiO_2_) MPs was collected by centrifugation and washed 3 times with distilled water. After washing, the MPs were dried overnight at 80 °C.

### Synthesis of Gadolinium (Gd)‐Doped BiOI (Gd‐BiOI) MPs

0.728 g of bismuth (III) nitrate pentahydrate (Bi(NO_3_)_3_∙5H_2_O, Sigma‐Aldrich) and 0.056 g of gadolinium (III) chloride hexahydrate (GdCl_3_∙6H_2_O, Sigma‐Aldrich) was dissolved in 20 mL ethanol, and 0.249 g of potassium iodide (KI, Sigma‐Aldrich) was dissolved in 40 mL deionized water separately. Then, KI solution was added drop‐wise into the Bi(NO_3_)_3_∙5H_2_O and GdCl_3_∙6H_2_O precursor solution under continuous stirring. The pH value of the mixture was adjusted to 7.0 by adding NH_3_∙H_2_O. Finally, the whole solution was stirred for 3 h at 80 °C. After cooling down, the orange precipitates were collected by centrifugation, washed several times with deionized water, and finally dried overnight at 80 °C.

### Synthesis of Indocyanine Green@Polydopamine Nanoparticles (ICG@PDA)‐Coated HNS‐MPs

BiOI or ZnO MPs were first dispersed into 50 mL of Tris buffer (10 mM, pH 8.5), and then added with 200 mg of dopamine hydrochloride (Sigma‐Aldrich). The mixture solution was stirred continuously for 6 h. The as‐synthesized PDA‐BiOI (or PDA‐ZnO) MPs were washed 3 times with deionized water. Next, the PDA‐MPs were dispersed into 50 mL Tris buffer containing the indocyanine green (ICG, TCI Chemicals) with 1.0 mg mL^−1^ concentration. The mixture was stirred continuously overnight, and the formed ICG@PDA‐MPs were washed with deionized water. The ICG@PDA‐MPs were stored in a fridge (< 4 °C) before use.

### Synthesis of Nanoporous Carbon (NPC) MPs

PI MPs were first kept at 350 °C inside a tube furnace for 1 h to complete imidization of PI and then pyrolyzed under flowing Ar at 900 °C for 1 h and activated by flowing NH_3_ at 900 °C for another 1 h to form NPC MPs. After cooling down to room temperature, the NPC MPs were collected.

### Acoustic Trapping and Manipulation Under Static or Dynamic Fluid Flow

A 2 MHz focused ultrasound (FUS) transducer (SU‐101, Sonic Concepts) was attached to the bottom of the water tank and connected with an RF amplifier (150A100C, Amplifier Research) powered by a function generator (AFG1022, Tektronix). The amplified signal was monitored by an oscilloscope (MDO4024C, Tektronix). The voltage input applied to the function generator was 200 mV_pp_ or 250 mV_pp_, and it was amplified 200 times through the amplifier. To record the motion of acoustic trapping and manipulation, a microscope (Leica) equipped with a high‐speed camera (Phantom MicroLab 140, Vision Research, Ametek Inc.) with a framerate of up to 5000 frames per second (fps) was used. Tygon tubes with different diameters (inner diameters of 500 µm, 3.18 mm, and 4.77 mm) and a microfluidic channel chip (µ‐Slide y‐shaped, ibidi) were employed to test the acoustic trapping of different sample particles or solutions. To induce a flow with a certain flow velocity, a syringe pump (kd Scientific) was used.

### Finite Element Simulations

The finite element simulations were carried out using the thermoviscous acoustics module in the frequency domain and the creeping flow model of Comsol Multiphysics 6.2 (COMSOL, Inc) paired with the Multiphysics Model for Acoustic Streaming. The existing simulation developed by Muller et al.^[^ ^52^ ^]^ for the acoustophoresis of particles immersed in a standing acoustic wave field to fit the experiments with a FUS transducer was customized. The transducer geometry and the tubing model with varying inner diameters of 500 µm, 3.18 mm, and 4.77 mm were developed. The tubing position was chosen to have the focal spot of the FUS transducer in the center of the tubing. For the simulations, a 3.5 µm‐diameter solid particle and HNS‐MP made of 33 nanosheets were defined individually. As a material, silica was chosen for all particles to gain comparable results and only catch changes induced by the difference in morphology. All particles were surrounded with water, and the tubing walls were made of polydimethylsiloxane (PDMS). The water was treated as inviscid fluid to simplify the Stokes drag due to computational limitations. The simulated flow speeds may therefore be higher than during the actual experiments. Here, The acoustic parameters of PDMS instead of Tygon because the parameters vary with the types of Tygon tubes used, and there is no data about the parameters available from the manufacturer. As a boundary condition, a perfectly matched layer for the water as the tubing and the transducer were immersed in water was defined. Based on the acoustic pressure measurement of the FUS transducer (Figure S4e, Supporting Information), an incident acoustic pressure wave with a pressure of 500 kPa in focus was defined. A sketch of the simulation geometry and a flow chart of the simulation can be found in Figure S9 (Supporting Information).

### Pressure Measurements

The setup consisted of a FUS transducer with a central frequency of 2 MHz (SU‐101, Sonic Concepts) or 500 kHz (H‐104G, Sonic Concepts) embedded into a customized glass tank with dimensions of 50 × 50 × 30 cm. The transducer was fixed to the side of the tank using double‐sided adhesive and connected to an amplifier (150A100C, Amplifier Research). A function generator (AFG 1022, Tektronix) attached to the amplifier generated the input for the amplifier including the input voltage and the frequency. To measure the output signal of the amplifier also an oscilloscope (MDO 4042C, Tektronix) was connected to it. To access the pressure applied by the transducer and to characterize the focal point, a needle hydrophone (HNA‐0400, Onda) was attached to an automated *x‐y‐z* stage using rods and a holder (Thorlabs). The hydrophone is placed in front of the transducer. The hydrophone was connected to an oscilloscope (MDO 4042C, Tektronix) to record the acquired signal. The whole process of data acquisition and focus finding was automated by a customized MATLAB code. A burst of US was sent with the transducer and the signal was recorded using the hydrophone. This procedure was repeated for several *x‐y‐z* positions. The acquired pressure map for different amplitude and frequency data at each specific point was used to extrapolate for other locations. Finally, the pressure at the focal point of the transducer was also measured for different input voltages. Using the calibration sheet of the hydrophone the measured voltage was transformed into pressure.

### Ultrasound (US) Imaging

B‐mode US imaging was performed using a commercial medical US imaging system (Vevo 3100 System, Fujifilm VisualSonics). The imaging was carried out by a US transducer (Model No. MX550D) operating within the frequency of 25–55 MHz and offering a scan depth of 15 mm. In order to conduct US imaging of different MPs (solid‐ or HNS‐) and SonoVue microbubbles, the samples were prepared using a cylindrical tubing with a diameter of 2 mm. For the MPs, solid‐ or HNS‐MPs with varying concentrations were dispersed into an agar aqueous gel (agar concentration of 1.4 wt.%). Subsequently, the agar gel‐MP mixtures were injected into the cylindrical tubes and allowed to solidify at room temperature. These cylindrical tubes, being filled with either MPs or SonoVue microbubbles, were then inserted into an agar phantom that featured several cylindrical voids (Figure S12a, Supporting Information). To ensure proper acoustic coupling and conduction of the US imaging, an ultrasound gel (Aquasonic clear) was applied atop the agar phantom. US imaging of Gd‐BiOI MPs injection into an *ex vivo* mouse tumor vessels was conducted using freshly deceased mice (within ≈6 h) having cancer tumors, which were provided by the Max‐Planck‐Institute for Biology of Ageing located in Cologne, Germany.

### Optical Coherence Tomography (OCT) Imaging

OCT imaging was performed with an OCT imaging system (TEL320C1 Spectral Domain OCT System, Thorlabs) with an input light source at the wavelength of 1300 nm. To obtain OCT signals of the different MPs or SonoVue microbubbles, the agar phantom containing the cylindrical tubes with samples inside aforementioned in US imaging was employed once again. The imaging was conducted at a medium sensitivity of 76 kHz, a refractive index of 1.00, and employing the Hann filter for the apodization window. The A‐scan averaging was set to 1, and the B‐scan averaging to 1 with a pixel size of 6.5 µm.

### Optoacoustic Tomography (OAT) Imaging

Multispectral optoacoustic tomography (MSOT) imaging was conducted employing a MSOT imaging system (inVision 512‐echo, iTheraMedical). For the acquisition of OAT signals from the various MPs or solution samples, the same agar phantom containing the cylindrical sample tubes, previously used for US and OCT imaging, was utilized. The OAT signal spectrum was obtained by scanning wavelengths across the range from 980 to 650 nm. OAT imaging of Au NP‐BiOI MPs injection into an *ex vivo* mouse peritoneal cavity was conducted using freshly deceased mice (within ≈6 h) provided by the Max‐Planck‐Institute for Biology of Ageing located in Cologne, Germany.

### Magnetic Resonance Imaging (MRI)

MRI imaging was carried out utilizing a 7 Tesla (7T) preclinical MRI scanner (BioSpec 70/30, Bruker). T1 and T2‐weighted MRI imaging of the agar gel, BiOI MPs, and Gd‐BiOI MPs employed the agar phantom samples containing dispersed MPs. For the MRI imaging of injected Gd‐BiOI MPs into ex vivo mice, the Fast Low‐Angle Shot (FLASH) mode with fat suppression was employed. The TR/TE parameters were set as either 500/6.6 ms or 500/9 ms (TR: repetition time, TE: echo time). The deceased mice utilized for MRI imaging were the same as those mentioned above.

### Cell Cytotoxicity Test

The human skin fibroblast cells, BJ (American Type Culture Collection), were cultured with high‐glucose Dulbecco's modified Eagle's medium (DMEM, Gibco) supplemented with 10% fetal bovine serum (Gibco) and 1% penicillin/streptomycin (Gibco) in a 75‐cm^2^ polystyrene cell culture flasks at 37 °C and 5% CO_2_. Cells were detached from the surface using trypsin (0.25 wt.%)/EDTA solution (Gibco) at 80% confluency. The cultured BJ fibroblast cells were seeded in a 96‐well plate with a black/clear bottom (Corning) at a concentration of 1 × 10^4^ cells well^−1^ the day before the experiment to allow the cells to attach to the bottom of the plate. The following day, the media of BJ fibroblast cells were changed with HNS‐MPs containing solutions prepared at different concentrations (50, 100, 250, 500, 750, and 1000 µg mL^−1^ in DMEM). After incubating the cells with different HNS‐MPs for 24, 48, and 72 h, the viabilities of BJ fibroblasts were tested at defined time intervals using the CellTiter‐Glo assay (Promega). The viability of treated fibroblasts is based on untreated fibroblasts, for which viability was assumed as 100%. All the experiments were performed in triplicates.

### Hemocompatibility and Thrombogenicity Measurements

Fresh mouse blood samples were collected in the animal facility of Tübingen University. Microparticles were sterilized with 70% ethanol and 1‐h UV light incubation, then air‐dried at the bottom of the cell culture wells. Whole blood samples were added to the cell culture wells containing the microparticles, and the cell culture plates were agitated for 15 min, 1200 RPM at room temperature. After the incubation, the blood samples and devices were used for luciferase assay and hematoxylin & eosin stainings. Four independent experiments were performed using fresh mouse blood samples for each test. Platelet aggregation was measured using the luminescence method in a plate reader (BioTek Synergy HTX, Agilent). The ATP release from activated platelets was measured using a luciferin‐luciferase reaction for the platelet activation test. In brief, this test used a ready‐to‐use Luciferin‐luciferase ATP measurement kit (CTG, CellTiterGlo Luminescent Cell Viability Assay, Promega). 100 µL CTG mix was added into the well containing the previously agitated samples for 15 min, 1200 RPM at room temperature before measuring platelet aggregation using luminescence. The whole blood sample with collagen‐I (100 µg mL^−1^) was used as a positive control, and a fresh whole blood sample incubated with phosphate‐buffered saline (PBS) was used as a negative control. Hematoxylin & eosin staining (ab245880, Abcam) were made according to the instructions of the commercial producer. The slides with thick blood smear samples embedded with xylene‐based mounting medium after hematoxylin & eosin staining. Six different light microscopy images were collected from each sample to quantify leukocytes and erythrocytes in different experimental groups.

### POUS System for Parallel OAT Imaging and FUS Trapping

For parallel imaging and FUS trapping of HNS‐MPs, a custom‐designed high‐resolution parallelized optoacoustic imaging and ultrasound (POUS) array system was employed that was used in the previous study.^[^ ^37^ ^]^ This array is composed of 512 individual transducer elements, each having a diameter of 2.5 mm. These elements were arranged in a spherical pattern on a shell with a curvature radius of 4 cm, spanning an angular extent of 140 degrees. The transducer elements were spaced apart by 3.2 mm in the elevational direction and 3.9 mm in the azimuthal direction. The array is interfaced with a custom‐designed multichannel electronics Data Acquisition (DAQ) system (Falkenstein, Mikrosysteme). The DAQ system utilizes class D amplifiers with a bandwidth of 15 MHz. These amplifiers were capable of driving all the transducer elements with digitally generated waveforms of variable durations. The voltage used for driving can be precisely adjusted within the range of 3.5 to 20 V_pp_. Additionally, the system allows for fine‐tuned transmission delay adjustments on a per‐channel basis with a time resolution of 5.5 ns. This high resolution surpasses the characteristic rise time at the emission frequency. Coupled with the spherical arrangement of the transducer elements, this feature minimizes grating lobes during emission, leading to a focus capability that closely approaches the diffraction limit. During transmission at a frequency of 3 MHz and with a driving voltage magnitude of 5 V_pp_, each individual transducer receives an electrical power supply resulting in an effective power of 0.1 W. This electrical power is then efficiently converted into acoustic power at an approximate efficiency of 30%. The same electronics employed in the system were adapted for the digitization of optoacoustic signals captured by the array elements. This digitization process occurs at a rate of 40 million samples per second (MS/s), and the acquired data is subsequently transmitted via an Ethernet port to a personal computer for storage and further postprocessing. During the imaging procedure pulse laser light (SpitLight, Innolas GmbH) with a repetition rate of 20 Hz and wavelength of 760, 800, and 850 nm was used.

### In Vivo Multi‐Trap Acoustic Maneuvering and 3D Imaging Within Blood Vessels

An in vivo experiment in a mouse was performed according to the national guidelines of the Swiss Federal Act on animal protection and was approved by the Cantonal Veterinary Office Zurich (ZH092/22). Animals were housed in individually ventilated cages inside a temperature‐controlled room, under a 12‐h dark/12‐h light cycle. Pelleted food (3437PXL15 and CARGILL) and water were provided ad libitum. The female Foxn1^nu^ nude mouse (5 weeks old) was anesthetized with isoflurane (3% during induction, 1.5% during the imaging) while under continuous monitoring of blood oxygen saturation, heart rate, and mouse body temperature (PhysioSuite, Kent Scientific, Torrington, CT). Mouse body temperature was kept within physiological range with a heating pad. The hair on the mouse was removed using shaving cream to ensure optimal ultrasound coupling. For precise adjustment of the mouse location, it was positioned on an *x‐y‐z* manual moving stage (Thorlabs). The near‐infrared light is delivered to the mouse femoral vein through an 8 mm diameter central aperture in the POUS system. After successful anesthesia of the mouse, 100 µL of 1 mg mL^−1^ ICG@PDA‐ZnO HNS‐MPs were injected into the tail vein as a bolus injection. To manipulate the particles inside the femoral vein, the mouse was moved using the *x‐y‐z* moving stage relative to the static POUS system. The mouse was euthanized immediately after the imaging procedure.

### Photocatalytic Water Flow Purification by HNS‐MPs

20 mL of Rhodamine B (RB, Sigma‐Aldrich) aqueous solution (concentration of 0.2 µM) was first prepared, then mixed with Au NP‐TiO_2_ MPs into 5.0 mg mL^−1^ concentration. The mixture solution was injected into the microfluidic channel chip (µ‐Slide y‐shaped, ibidi) and circulated at a velocity of 278 mm s^−1^ using a syringe pump. After the trap of Au NP‐TiO_2_ MPs was formed in RB solution flow by a 2 MHz transducer (SU‐101, Sonic Concepts, U.S.A.), ultraviolet (UV, 365 nm) and visible (515 nm) light were irradiated to the trap by LED light sources (365 nm: M365L4, Thorlabs and 515 nm: 3W515525s, Avonec) equipped with a collimator lens (SM2F32‐A, Thorlabs) using an LED driver (DC4104; Thorlab). A USB microscope camera (Toolcraft) was set from the top of the experimental setup and monitored the color change of the RB solution. During the light irradiation, 0.5 mL of the RB solution underwent the UV–Vis spectrum measurement every 20 min.

### Doxorubicin Loading into HNS‐MPs

Doxorubicin hydrochloride (DOX, Sigma‐Aldrich, suitable for fluorescence) dissolved in dPBS was first prepared in a concentration of 0.5 mg mL^−1^. Before agitation with different MPs, the UV‐Vis spectrum of the initial DOX solution was obtained. After 24 h of stirring with MPs to facilitate DOX coating or absorption, the suspension underwent centrifugation, and a UV–Vis spectrum of the supernatant was measured to quantify the DOX loading efficiency. For fluorescent microscopy imaging, the DOX‐loaded MPs were drop‐casted on glass slides and imaged through a FITC filter (MDF‐FITC, Emission wavelength of 475 nm, Thorlabs) using a fluorescent microscope (Nikon Eclipse Ti‐S).

### Driving Forces of Focused Ultrasound Enabling Particle Trapping

The main driving forces for acoustic manipulation were the primary acoustic radiation force, *F_R_* and the acoustic streaming‐induced drag force, *F_AD_*. A single particle with radius, *a* immersed in a sound field will experience an acoustic radiation force, *F_R_* occurring due to pressure differences around the particle caused by the scattering of sound waves along the particle surface. If the focus area of the simulated FUS field as a quasi‐standing wave in 1D was treated, the radiation force on a particle is given by:^[^ ^50^ ^]^

(1) $$F_{R}=4\pi \phi a^{3}qE_{ac}sin\left(2qy\right)$$

(2) $$\phi =\frac{1}{3}\left(\frac{5\frac{\rho_{p}}{\rho_{m}}-2}{2\frac{\rho_{p}}{\rho_{m}}+1}-\frac{\kappa_{p}}{\kappa_{m}}\right)$$

where *q*, *E_ac_*, and *y* are the wave number, acoustic energy density, and particle position in *y–*axis, respectively. The acoustophoretic contrast factor *ϕ* depends on the fluid and particle density. ρ_*m*_ and ρ_*p*_ as well as the fluid and particle compressibility κ_*m*_ and κ_*p*_.

Additionally, it will experience a drag force, *F_AD_* arising from acoustic streaming. Acoustically induced streaming can be divided into 2 different types based on their origin: bulk acoustic streaming and boundary streaming. Bulk acoustic streaming originates from the dissipation of acoustic fields inside the medium. In curved geometries, acoustic streaming can exhibit complex flow patterns due to the interplay between viscous boundary layers and the curvature of the fluid domain. While the overall bulk streaming remains incompressible, local flow behavior influenced by pressure gradients in curved channels may exhibit compressible‐like features, distinct from straight channel streaming. On the other hand, boundary streaming is caused by the viscous boundary layer near rigid walls, causing viscous damping of the acoustic field. This boundary layer is caused by shear and Reynold's stress building up in this region due to acoustic‐solid interaction. The thickness, δ of this boundary layer can be calculated by:^[^ ^51^ ^]^

(3) $$\delta =\sqrt{\frac{2\;\nu_{0}}{\omega }}$$

where ν_0_ and ω are the kinematic viscosity of the medium and the angular frequency, respectively. Using Equation 3, in the case of a sound field applied by a 2 MHz focused transducer in water at room temperature, got δ  =  0.32 µm.^[^ ^52^ ^]^ Given the large difference between this δ value and the inner tubing diameters of 500 µm, 3.18 mm, and 4.77 mm, the inner boundary streaming part (“Schlichting streaming”) was therefore neglected and only consider the outer boundary component (“Rayleigh streaming”) as the particles were likely to be far away from this boundary layer.

The overall streaming velocity in the fluid is composed of 2 parts: an incompressible velocity component that originates from boundary streaming and a compressible velocity component that results from bulk acoustic streaming. The incompressible component occurs near the boundaries, where the fluid flows steadily along surfaces without significant changes in density due to viscous effects. In contrast, the compressible component occurs in the bulk of the fluid, where the fluid's density can change as it moves in response to pressure gradients established by the acoustic waves. As mentioned above, the incompressible velocity from the boundary streaming can be neglected beyond the boundary layer.^[^ ^51^ ^]^ Therefore, the acoustic streaming‐induced drag force *F*_*AD*_on a particle can be calculated by:^[^ ^53^ ^]^

(4) $$F_{AD}=6\pi \eta a\left(\langle v\rangle -u\right)$$

where *η*, *a*, *v* and *u* are dynamic viscosity of the medium, the particle radius, the streaming velocity and the particle velocity, respectively. The design space to change *F*_*AD*_based on particle parameters is therefore limited to the radius of the particle [Equation 4]. In contrary, *F_R_* offers a wider range of parameters, which are the particle radius as well as the particle compressibility and density [Equations 1 and 2].

In more general terms, the acoustophoresis of a particle can be described by the Gor`kov potential *U*:^[^ ^30^ ^]^

(5) $$U=\frac{\left(4\pi a^{3}\right)}{3}\left(\frac{1}{2}f_{1}{\left|p\right|}^{2}-\frac{4}{3}f_{2}{\left|v\right|}^{2}\right)$$

The acoustic contrast factors *f*_1_ and *f*_2_ depend on the properties of the particle and the surrounding fluid, respectively. The acoustic radiation force *F_R_* on a particle is given by the negative gradient of the Gor'kov potential. Therefore, particles were attracted toward minimal Gor´kov potential regions.

## Conflict of Interest

The authors declare no conflict of interests.

## Author Contributions

D.W.K. and P.W. contributed equally to this work. D.W.K., P.W., and M.S. conceived the project and designed the experiments. D.W.K. and P.W. performed the experiments and contributed the data analysis and management. H.E. contributed to the animal experiments. E.Y. was involved in biocompatibility tests. J.Z. contributed to the magnetic resonance imaging experiments. A.B. was involved in result discussion. M.S. and D.R. supervised the study. D.W.K. and P.W. wrote the main manuscript, and all authors contributed to the manuscript editing.

## Supporting information



Supporting Information

Supplementary Movie 1

Supplementary Movie 2

Supplementary Movie 3

Supplementary Movie 4

Supplementary Movie 5

Supplementary Movie 6

Supplementary Movie 7

Supplementary Movie 8

Supplementary Movie 9

## Data Availability

The data that support the findings of this study are available from the corresponding author upon reasonable request.
