# Supplementary material for: Hierarchical Nanostructures as Acoustically Manipulatable Multifunctional Agents in Dynamic Fluid Flow
Source: Adv Mater. 2024 Oct 14;36(50):2404514. doi: 10.1002/adma.202404514 (PMC11636169; doi:10.1002/adma.202404514)
Supplement: Supplementary file 1 — Supporting Information [file ADMA-36-2404514-s008.pdf]

ADVANCED MATERIALS

Supporting Information

for *Adv. Mater.*, DOI 10.1002/adma.202404514

Hierarchical Nanostructures as Acoustically Manipulatable Multifunctional Agents in Dynamic Fluid Flow

Dong Wook Kim, Paul Wrede, Hector Estrada, Erdost Yildiz, Jelena Lazovic, Aarushi Bhargava, Daniel Razansky and Metin Sitti**

Supporting Information

Hierarchical Nanostructures as Acoustically Manipulatable Multifunctional Agents in Dynamic Fluid Flow

Dong Wook Kim, Paul Wrede, Hector Estrada, Erdost Yildiz, Jelena Lazovic, Aarushi Bhargava, Daniel Razansky, Metin Sitti**

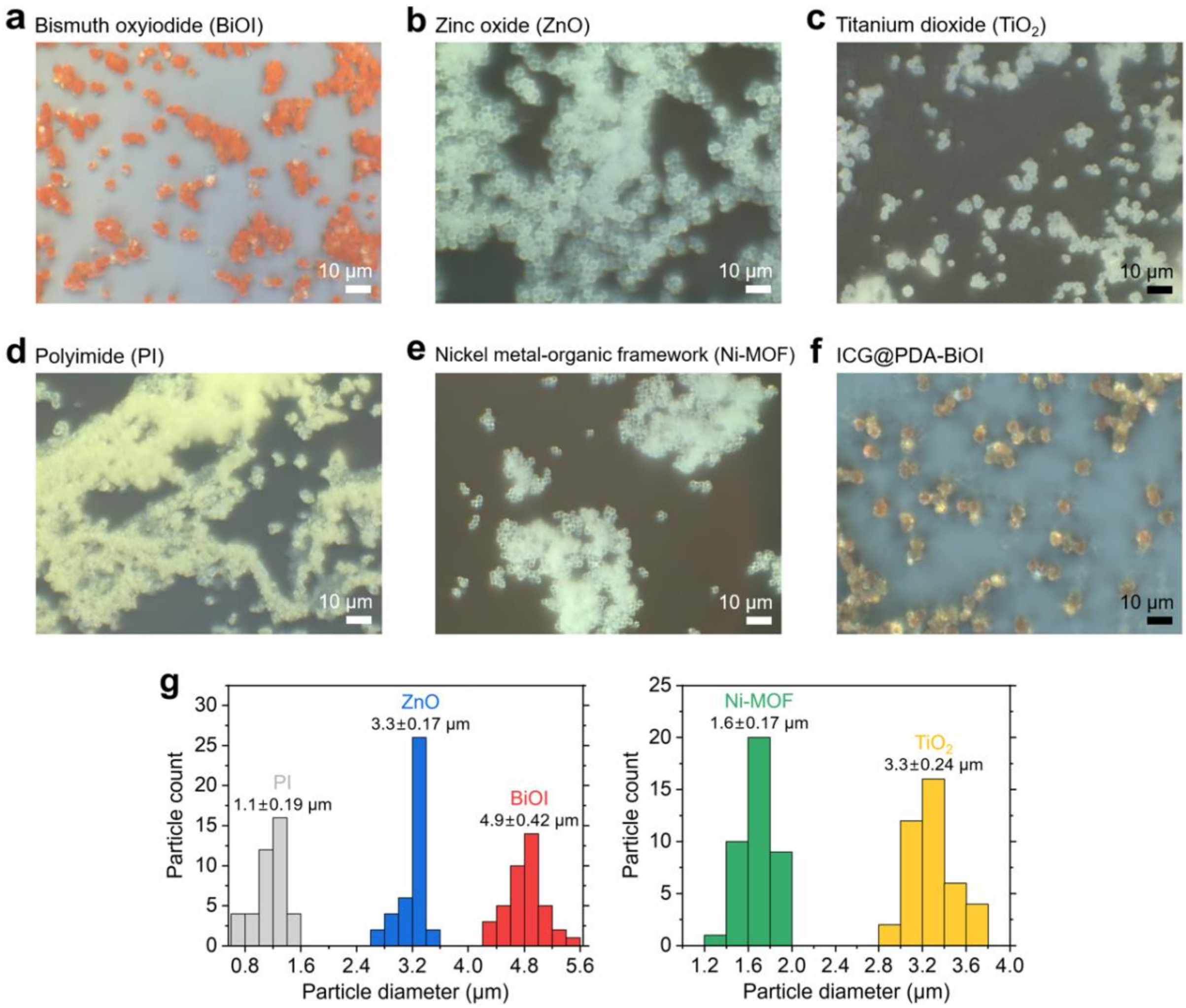

Figure S1. Size distribution of the five different HNS-MPs. a−f) Optical microscopy images of the five HNS-MPs: BiOI, ZnO, TiO_2, PI, Ni-MOF (a−e), and ICG@PDA-BiOI MPs (f). g) Histogram depicting the size distribution of the five HNS-MPs. The diameter of a total of 40 MPs was measured in scanning electron microscopy (SEM) images (See Figure S2) for each HNS-MP.

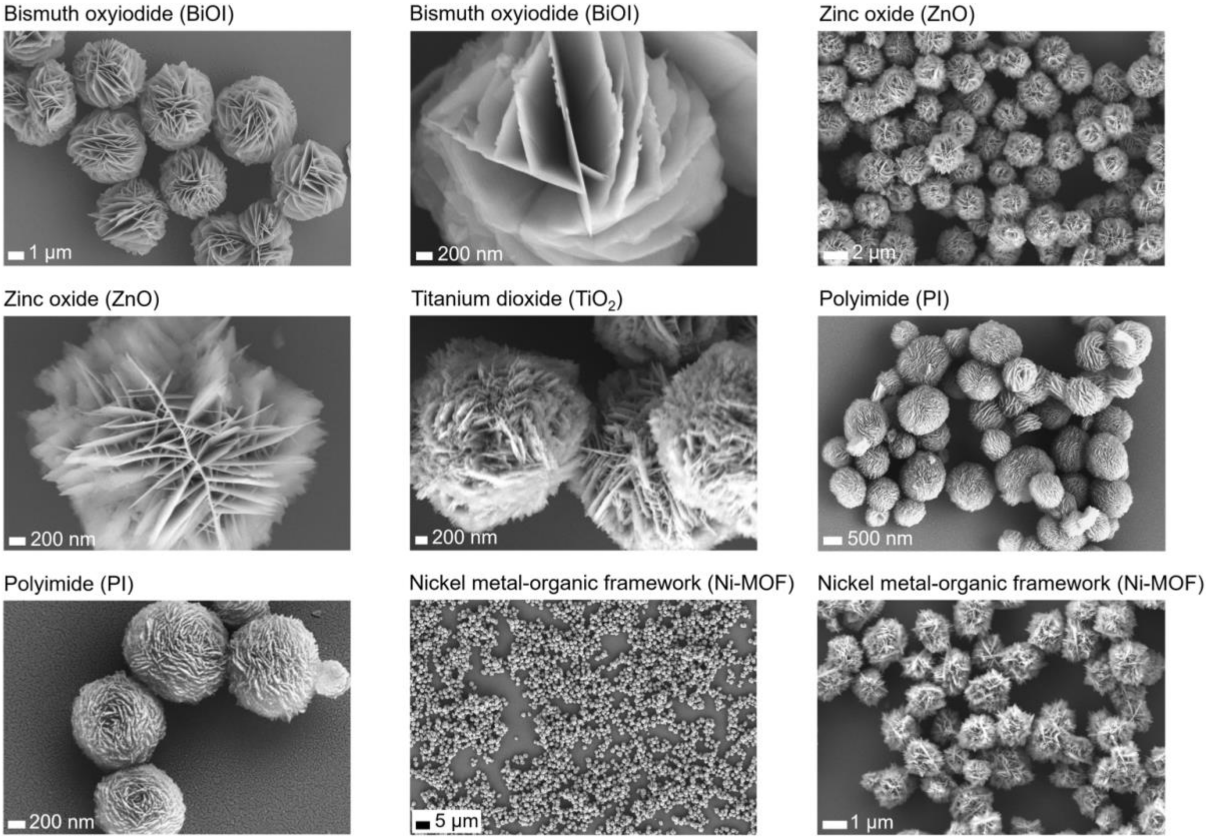

Figure S2. Morphologies of the five different HNS-MPs obtained by scanning electron microscopy (SEM).

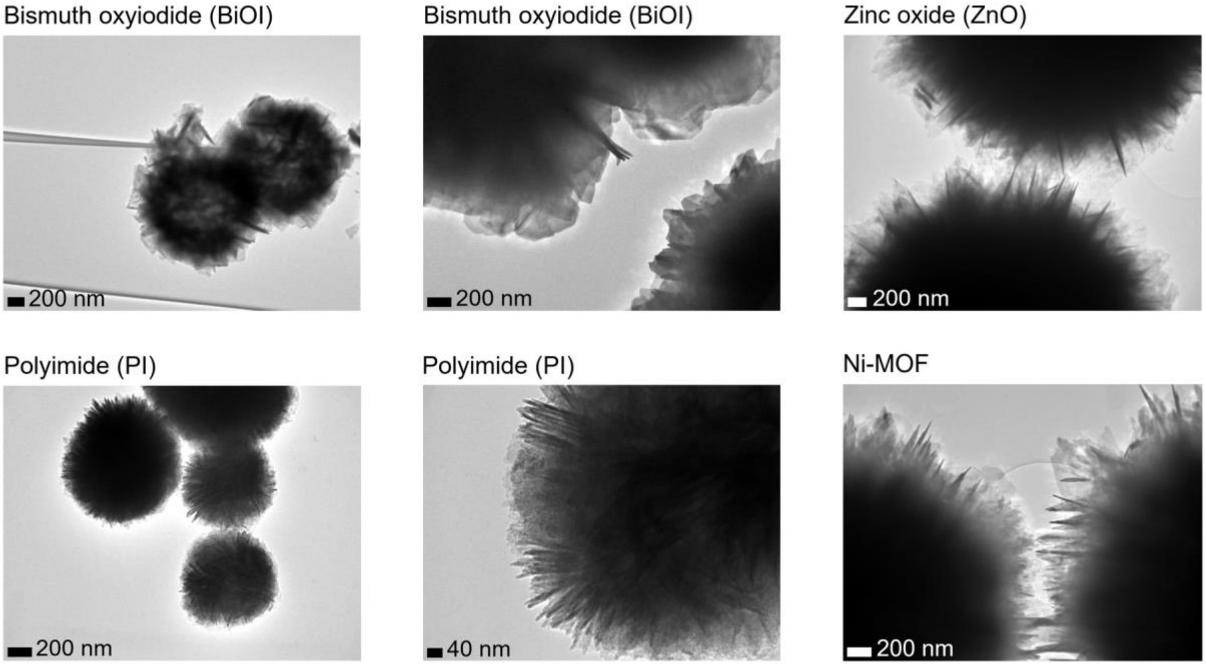

Figure S3. Morphologies of the different HNS-MPs obtained by transmission electron microscopy (TEM).

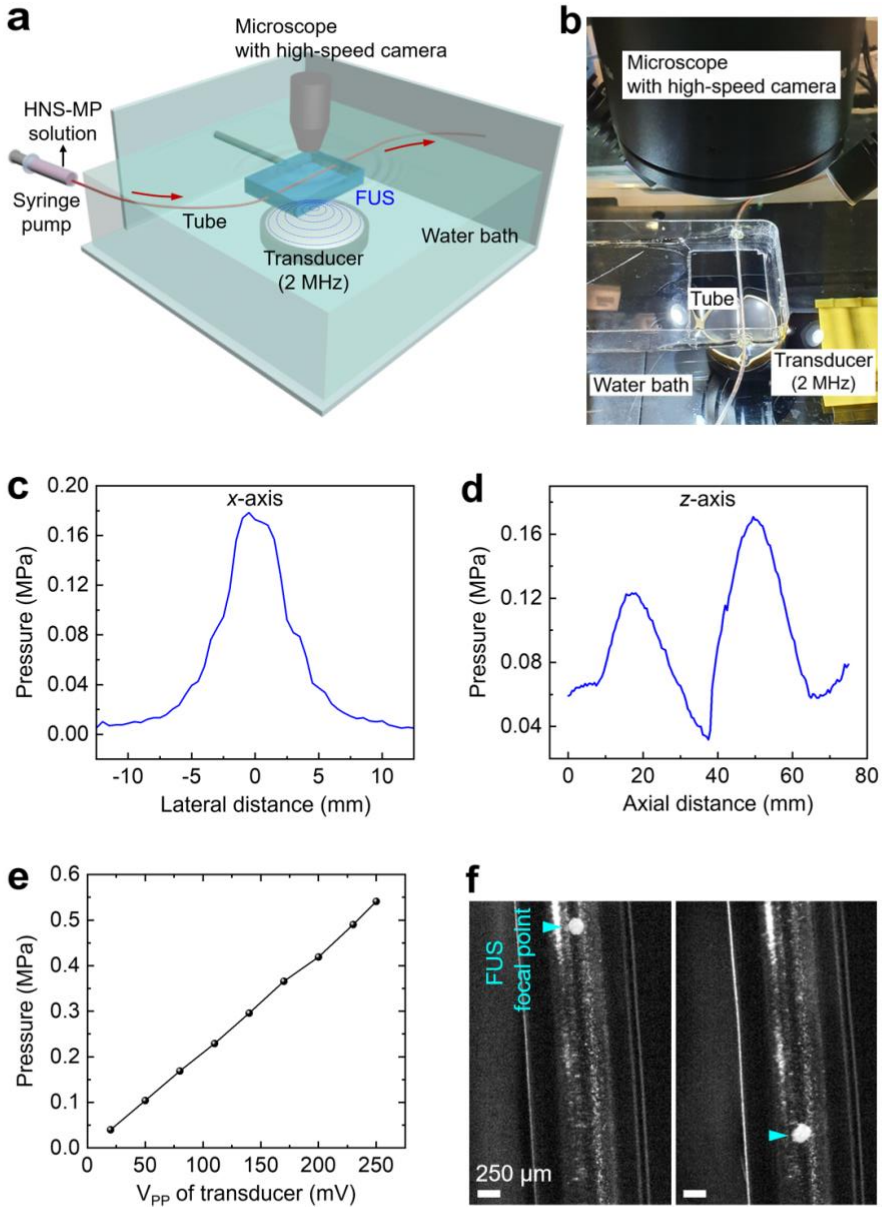

Figure S4. Experimental setup for testing acoustic trapping and manipulation in fluid flow. a,b) Schematic illustration (a) and photograph (b) of the setup for the acoustic trapping experiment using tubes. Tygon® tubes with various diameters (inner-diameters of 500 µm, 3.18 mm, and, 4.77 mm) and microfluidic channels were employed for the experiment. Acoustic trapping tests were conducted using a focused ultrasound (FUS) wave generated from a 2 MHz transducer were monitored and recorded by a top-mounted microscope equipped with a high-speed camera. c,d) Hydrophone measurement of the acoustic pressure profile from the 2 MHz FUS transducer by changing the distance between the hydrophone and the transducer in the lateral (*x*-direction) (c) and axial (*z*-direction) (d) directions. In the measured axial pressure profile, the first peak comes from the near-field diffraction effect of the FUS, while the second, larger peak corresponds to the FUS focus. e) Hydrophone measurement of the acoustic pressure at the focal point versus applied voltage of the 2 MHz transducer. f) Top-view of BiOI MPs trapped and manipulated inside the 500 µm-diameter tube while the FUS was driven. The trapped BiOI MPs moved inside the tube as the FUS focal point (indicated by cyan triangles) shifted.

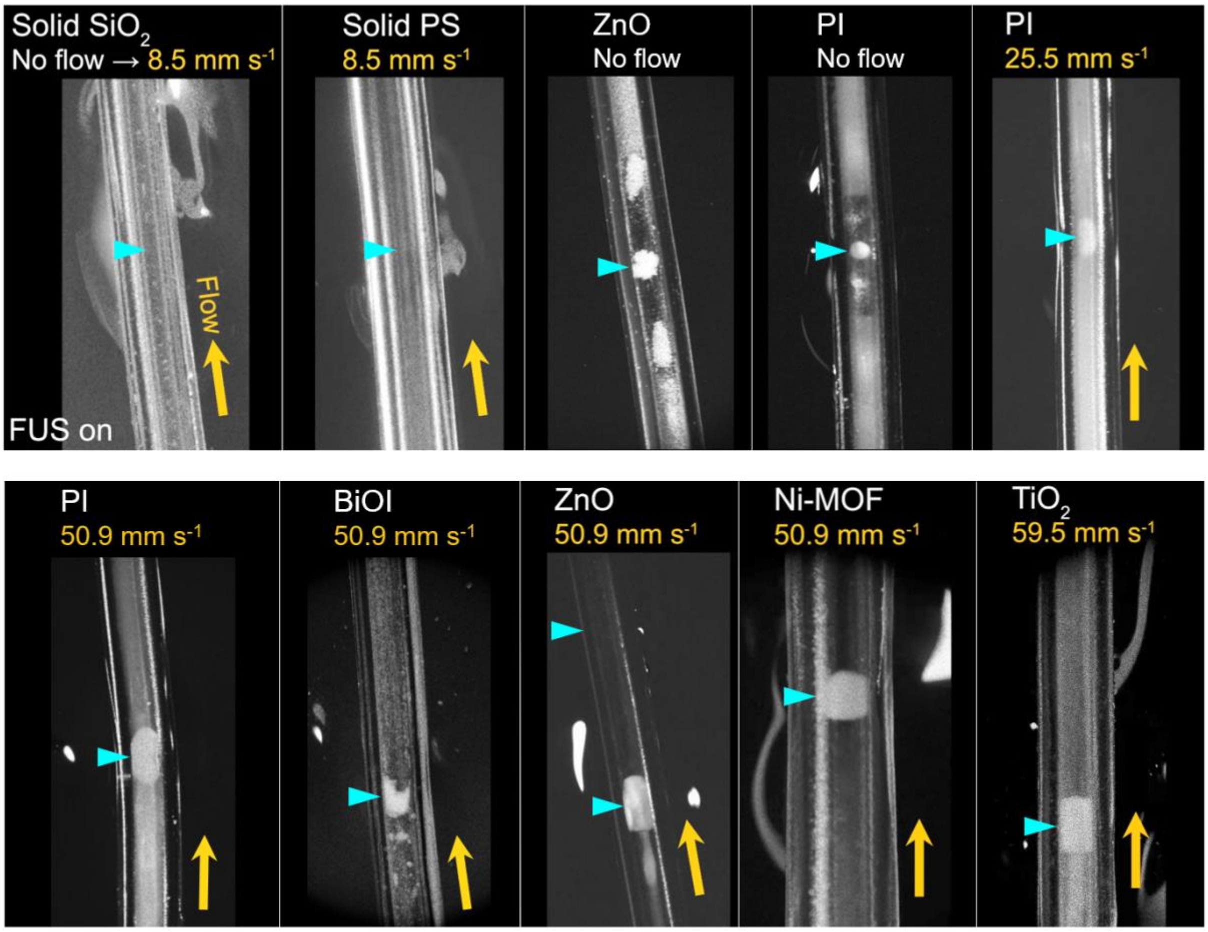

Figure S5. Acoustic trapping and manipulation test in fluid flow: Comparison between solid MPs and various HNS-MPs. Top-view of various particles flowing in the 500 µm-diameter tube under the activation of FUS (2 MHz, 200 mV_{pp}, 0.42 MPa). The cyan triangle marks the focal point of FUS and the yellow arrow indicates the direction of water flow. The solid SiO_2 (2 µm-diameter) or polystyrene (PS, 3 µm-diameter) MPs were able to be trapped when there was no flow, but under dynamic flow conditions, they could not be trapped by FUS. Conversely, not only in static flow but also in the flow velocity range of 8.5 to 59.5 mm s^{-1}, the five different HNS-MPs were trapped at the FUS focal point position and moved within the tube as the focal point position was shifted.

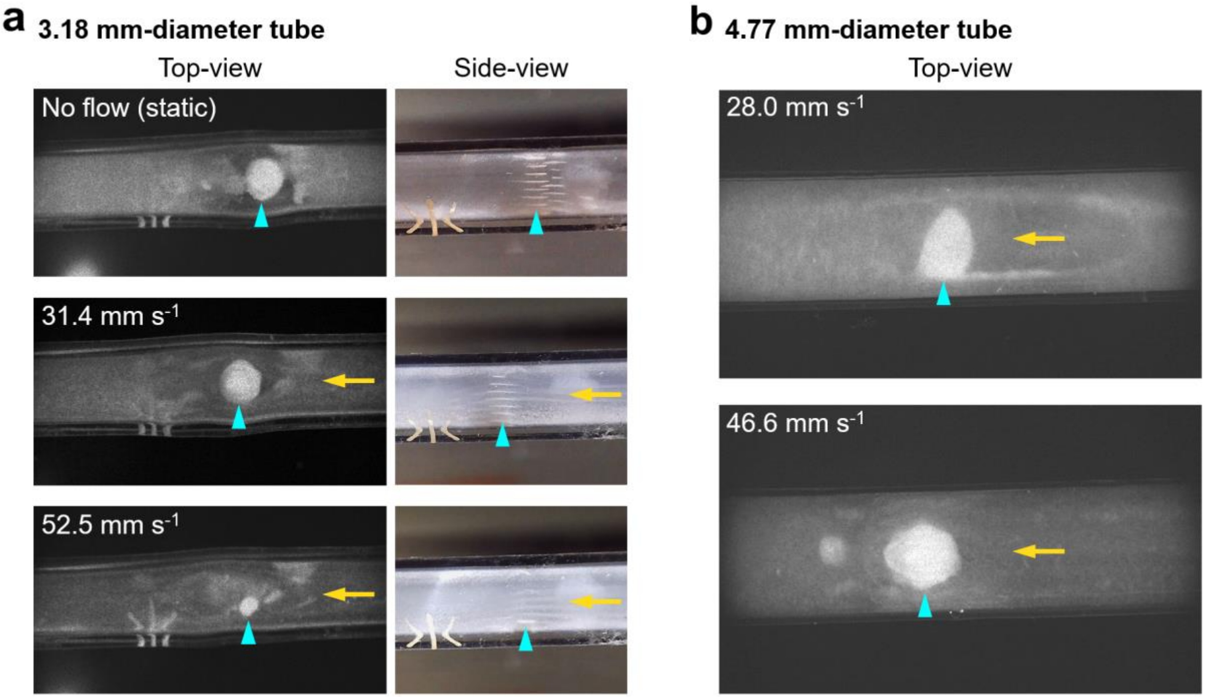

Figure S6. Acoustic trapping of HNS-MPs in larger tubes under fluid flow. a) Top- and side-views of the ZnO MPs trapped within a 3.18 mm-diameter tube at varying water flow velocities. In the 3.18 mm-diameter tube, stable trapping of ZnO MPs with multiple 2D traps was observed under water flow velocities up to 47.2 mm s^{-1}. However, as the flow velocity increased to 52.5 mm s^{-1}, The traps located near the center of the tube flowed away by the flow, whereas the trap at the tube's edge remained stable. This phenomenon occurs because the flow adjacent to the edge of the tube is slow, while the flow near the center is fast, in accordance with Hagen-Poiseuille law. b) Top-view of TiO_2 MPs trapped within a 4.77 mm-diameter tube, subject to different water flow velocities. The cyan triangle marks the focal point of FUS and the yellow arrow indicates the direction of water flow.

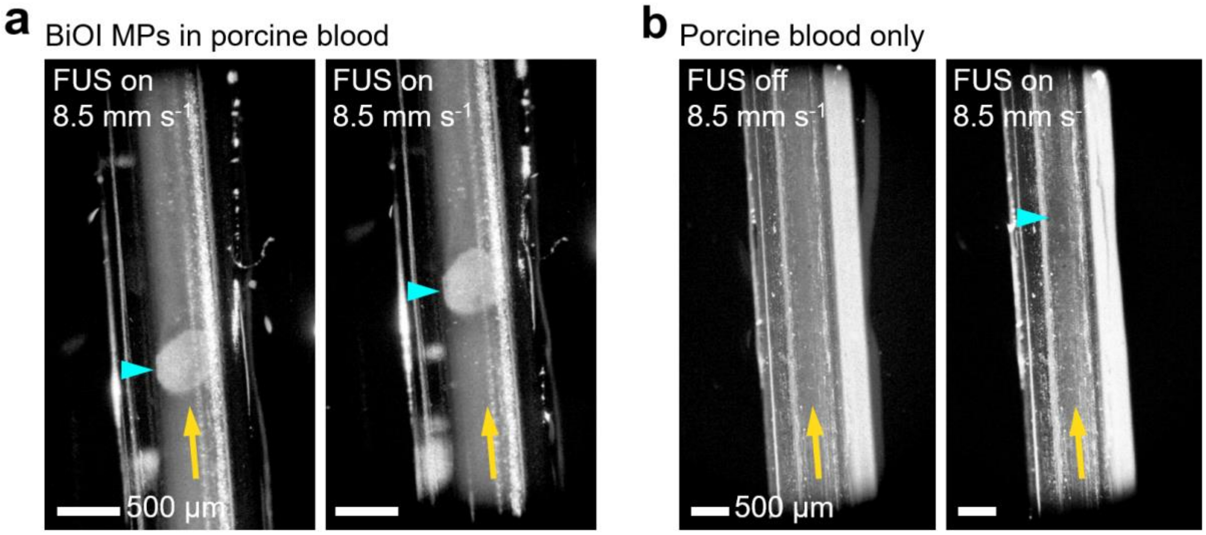

Figure S7. Acoustic trapping and manipulation of HNS-MPs in porcine blood flow. a) Microscopy images showing the manipulation of trapped BiOI MPs within a tube (500 μm-diameter) under porcine blood flow at a velocity of 8.5 mm s^{-1}. b) Microscopy images showing the change of porcine blood flow within the tube upon FUS activation. Upon activating the FUS, we observed the porcine blood being displaced both upward and downward in the tube from the FUS focal point. The cyan triangle marks the focal point of FUS and the yellow arrow indicates the direction of blood flow.

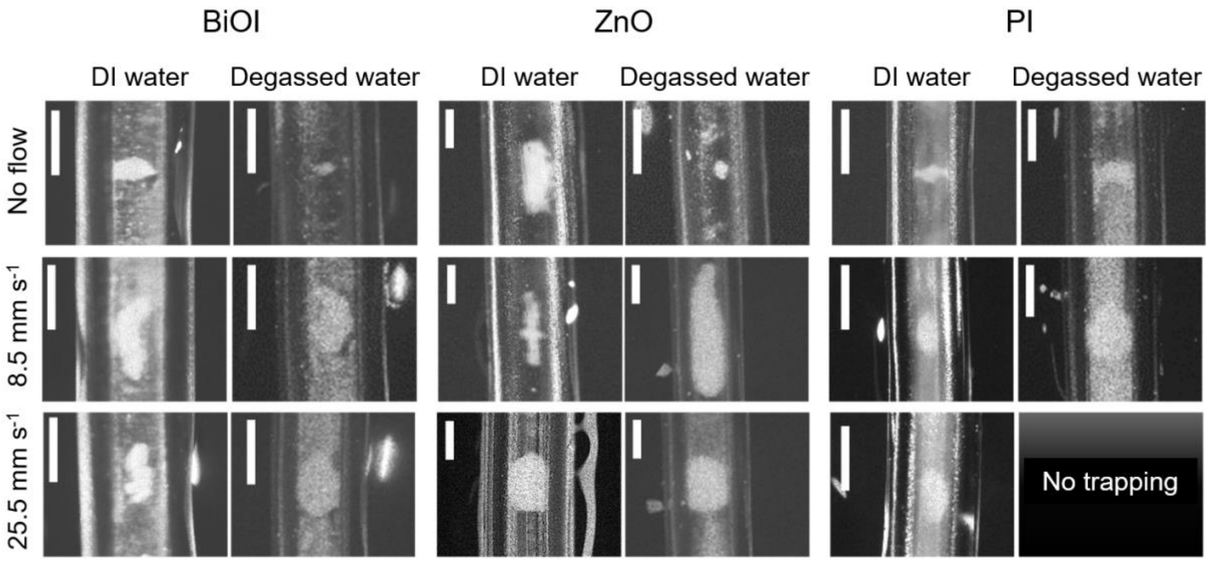

Figure S8. Acoustic trapping of HNS-MPs in regular water and degassed water. Comparative microscope images of trapped PI, ZnO, BiOI HNS-MPs under various flow conditions in regular deionized water and degassed water. The particles are trapped inside a 500 µm inner-diameter tubing using a 2 MHz FUS. The scale bars correspond to 500 µm.

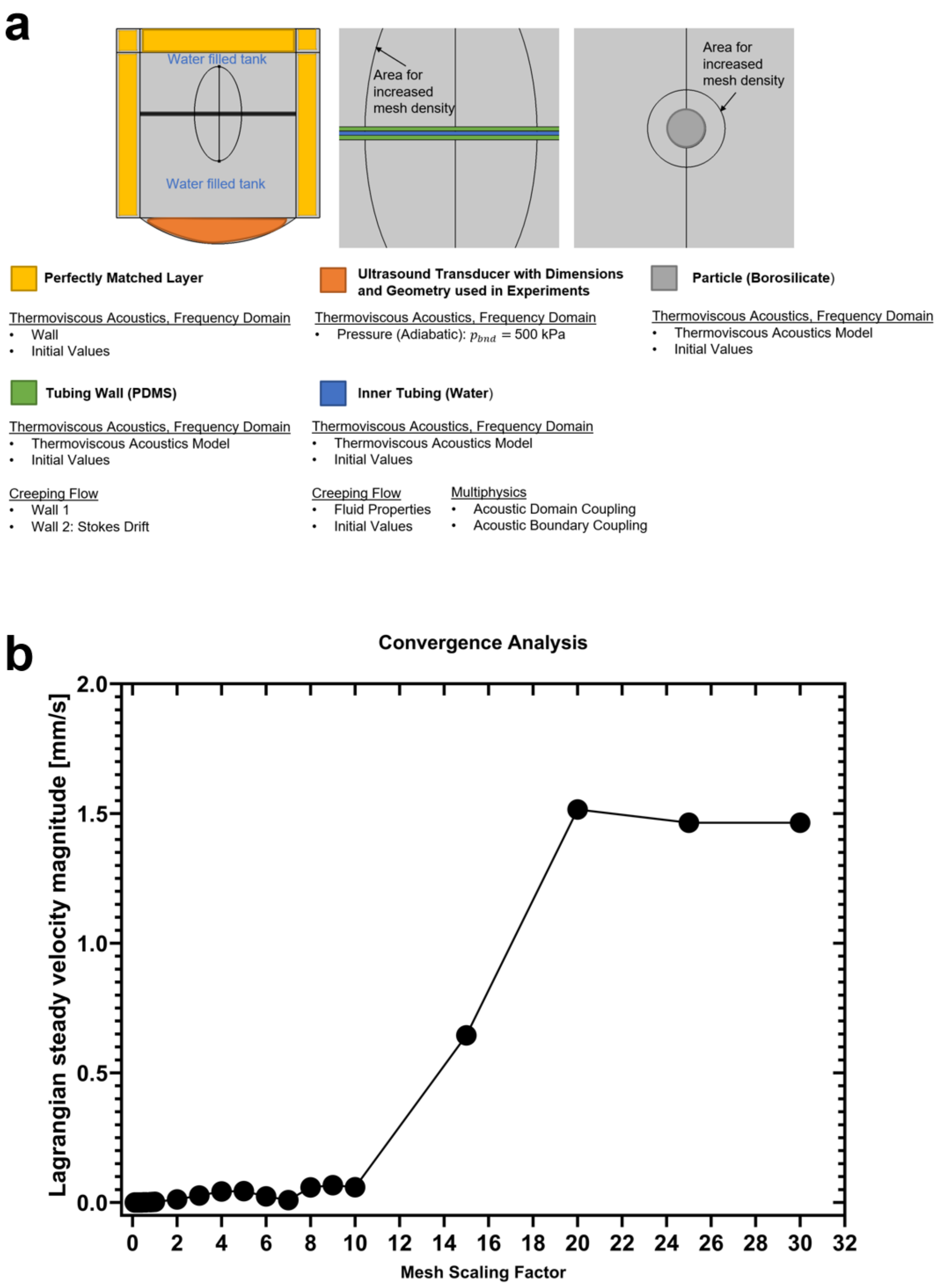

Figure S9. Summary of COMSOL 6.2 finite element setup and convergence. a) Simulation geometry with assigned physics and boundary conditions for simulations in COMSOL 6.2. b) Convergence plot for the Lagrangian steady velocity magnitude at a distance of 3.5 µm from the particle surface. The mesh used for our simulations has a mesh scaling factor of 1. A mesh scaling factor of 20 refers to a mesh 20 times larger than the one we used during simulations.

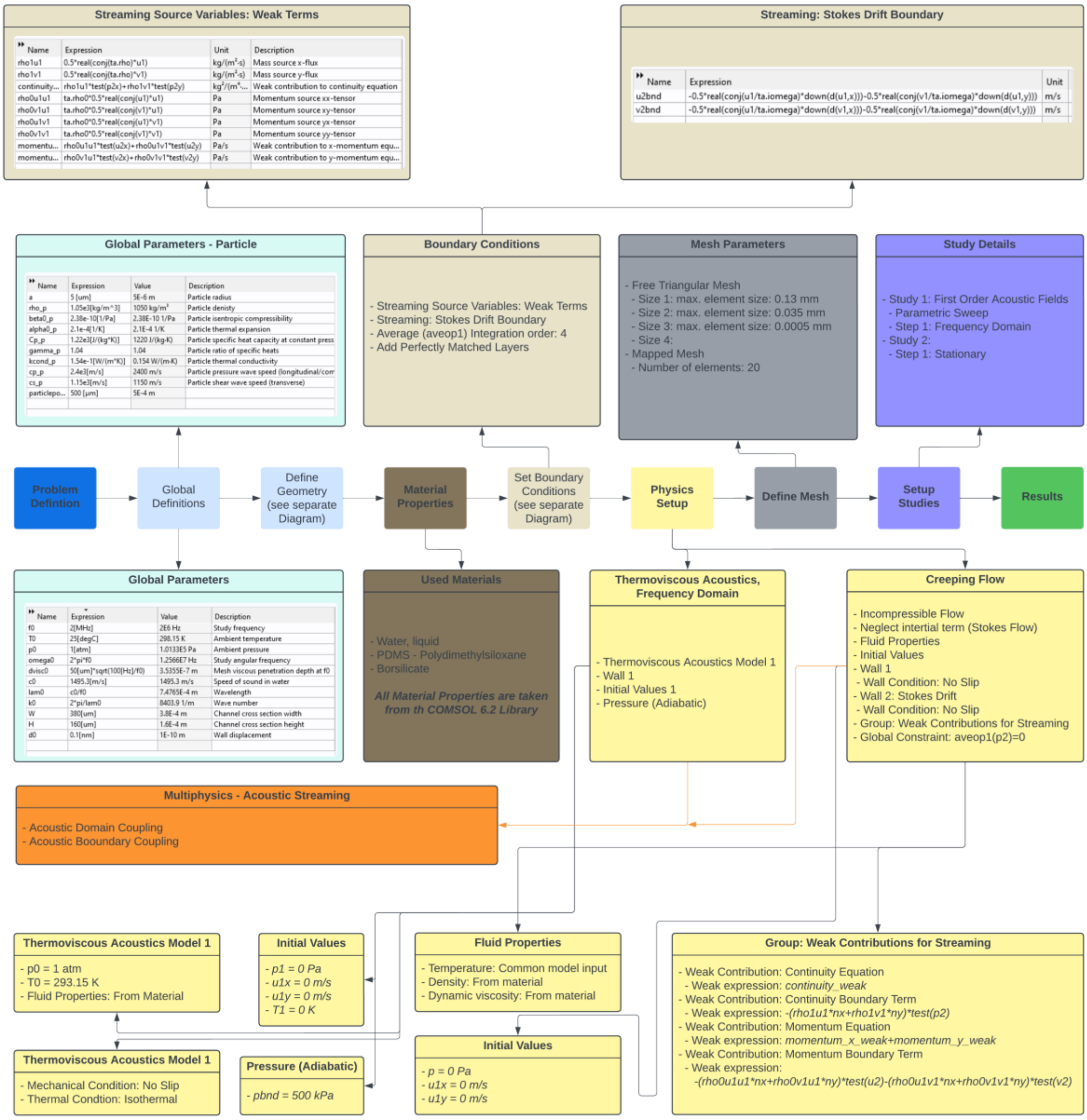

Figure S10. Simulation geometry and flow chart with used parameters for COMSOL 6.2 finite element simulations

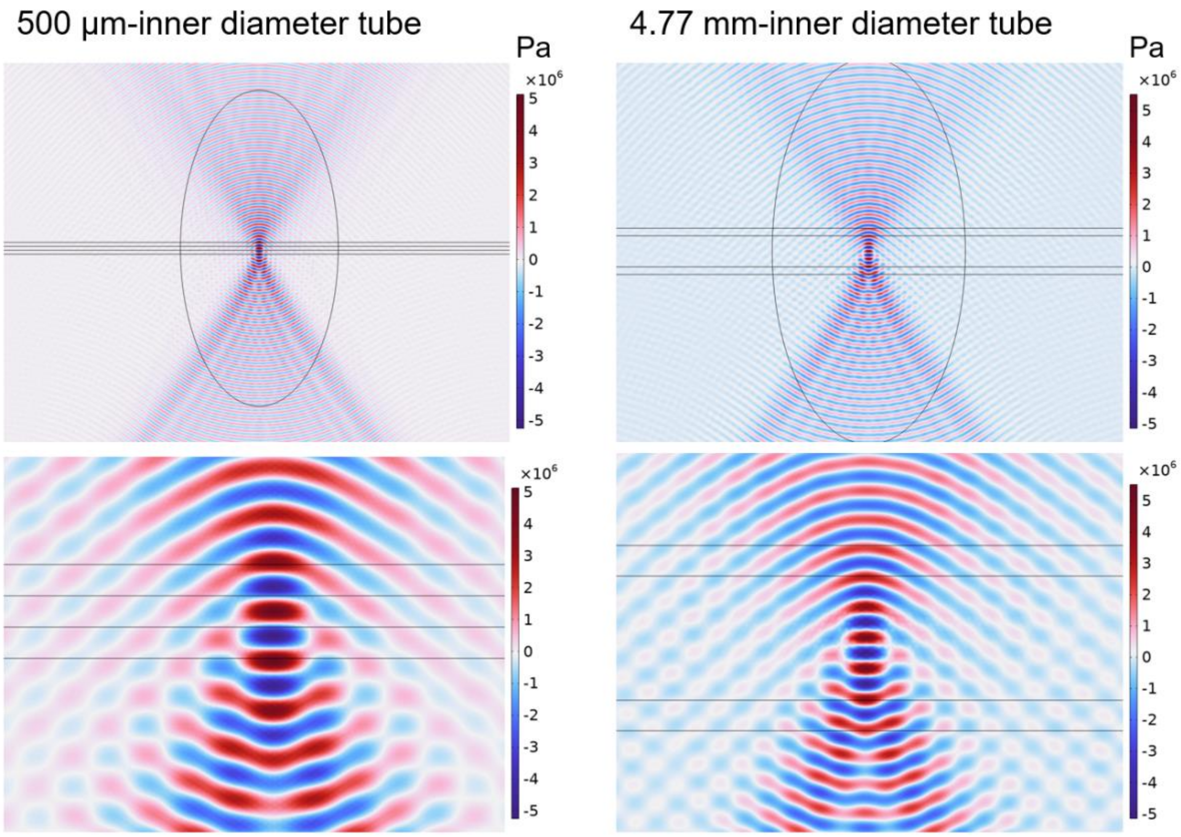

Figure S11. Simulated pressure field in 500 µm and 4.77 mm inner-diameter tubes applied from a 2 MHz FUS transducer. 2D depiction of the pressure, in frequency domain, applied by a 2 MHz FUS transducer as used during experiments. The acoustic wave is travelling through water into the tubes.

**Material of HNS-MPs**	**Average diameter of MPs**	**κ at 2 MHz**
PI	1.1	0.004
Ni-MOF	1.6	0.006
ZnO	3.3	0.013
TiO_2	3.3	0.013
BiOI	4.9	0.020

Table S1. Calculation of a dimensionless parameter κ for different HNS-MPs in our study.

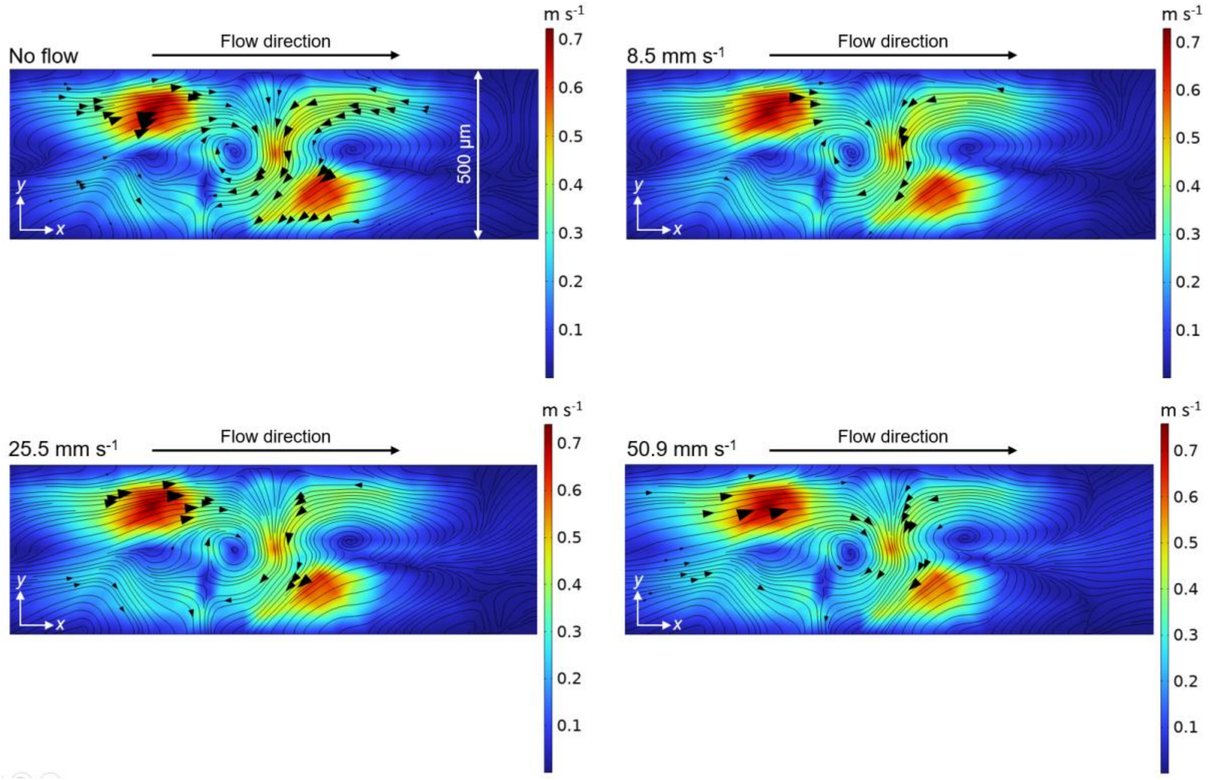

Figure S12. Acoustic streaming patterns simulated inside the 500 μm inner-diameter tube at different water flow velocities. The acoustic streaming flow within the tube is caused by the 2 MHz FUS applied from the transducer. The tubing is filled with water and assumed to be inviscid. The water flows from the left to right side.

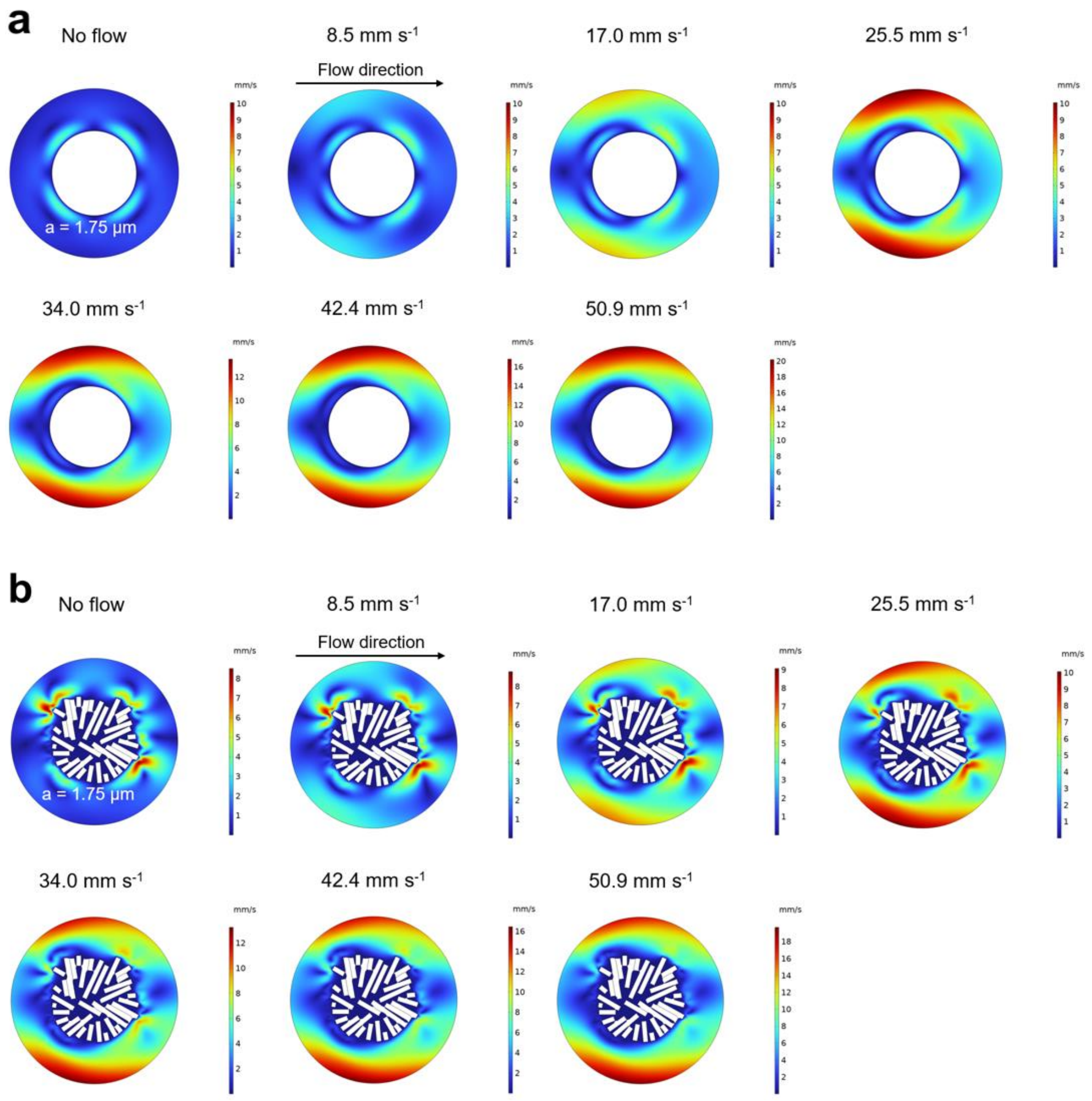

Figure S13. Secondary acoustic streaming around solid MP (a) and HNS-MP (b) and under static condition and with different fluid flows. The flow was applied from the left to right. Both particles with a radius (*a*) of 1.75 μm are positioned at the FUS focal point.

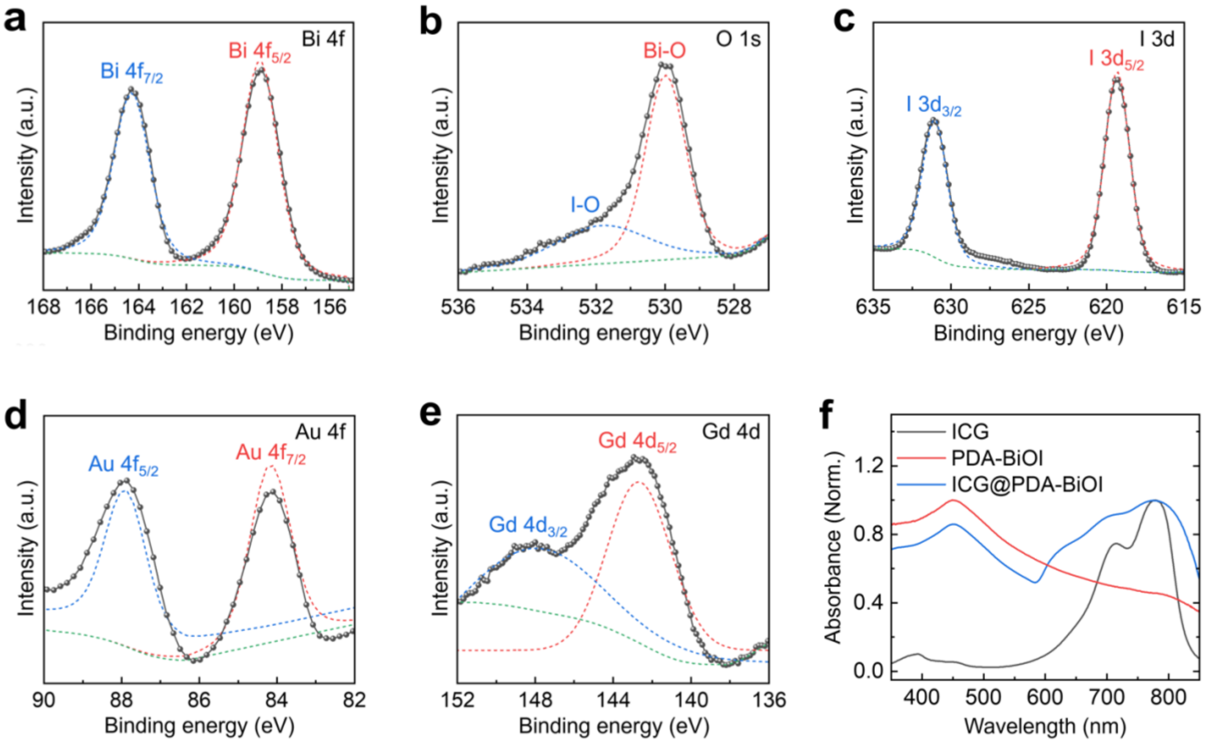

Figure S14. Characterization of the functionalized HNS-MPs. a−c) X-ray photoelectron spectroscopy (XPS) spectra of the BiOI MPs obtained at Bi 4f (a), O 1s (b), and I 3d (c). d) Au 4f XPS spectrum of the Au NP-BiOI MPs. e) Gd 4d XPS spectrum of the Gd-BiOI MPs. f) UV-Vis absorption spectrum of free ICG solution (50 mg mL^{-1} in water), PDA-BiOI MPs, and ICG@PDA-BiOI MPs.

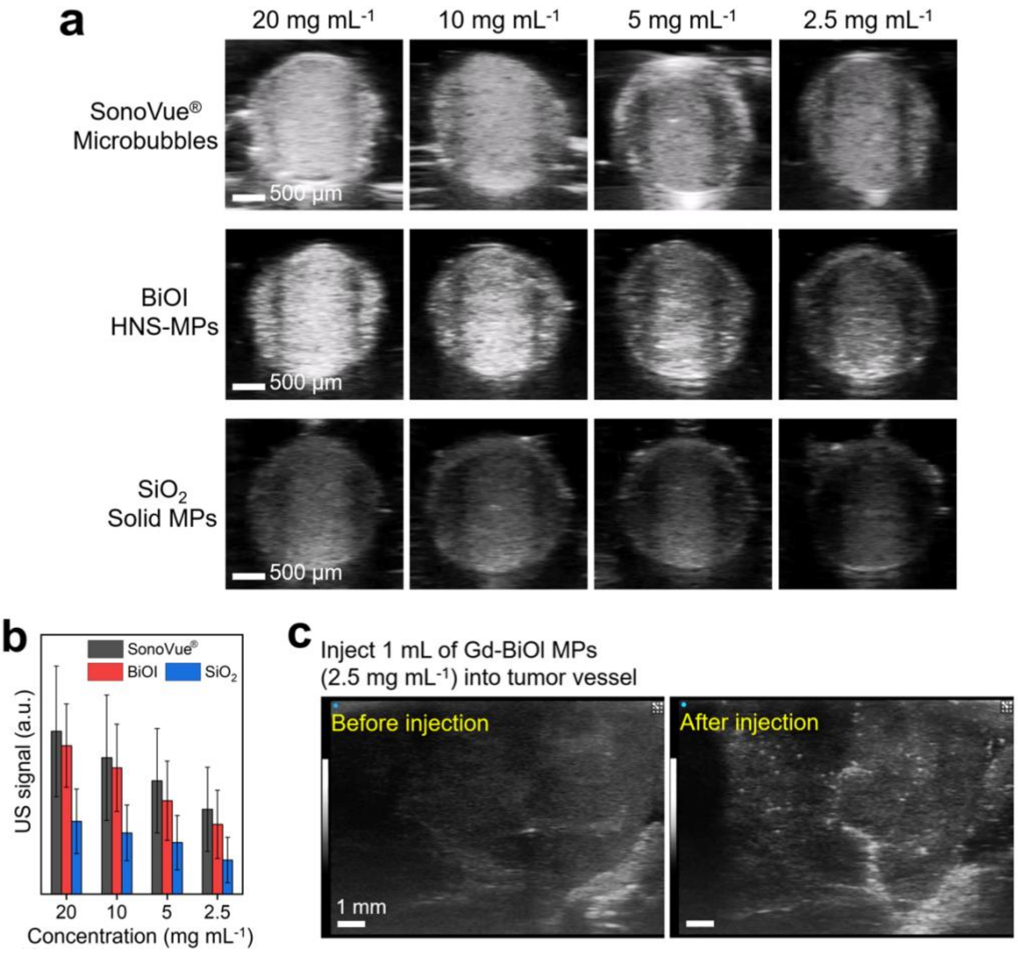

Figure S15. Ultrasound imaging (US) of HNS-MPs and signal intensities comparison between SonoVue® and solid SiO_2 MPs. a,b) US signal comparison from SonoVue®, BiOI HNS-MPs, and solid SiO_2 MPs samples of different concentrations. US signal intensities were obtained from each sample distributed in a cylindrical agar phantom tube. To conduct US imaging, the sample tubes were inserted into an agar gel phantom and ultrasound gel was placed on top of the agar phantom (See Experimental Section for more details). c) US imaging of Gd-BiOI MPs injected into *ex vivo* mouse cancer tumor that clearly visualized blood vessel distribution of the tumor.

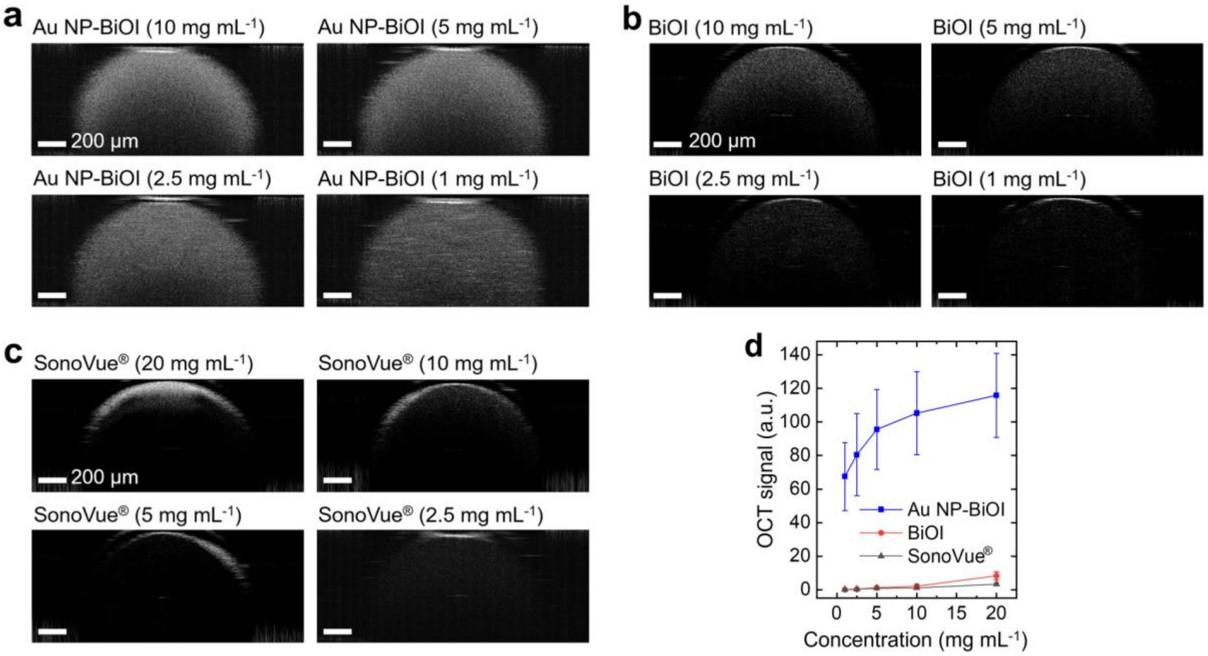

Figure S16. Optical coherence tomography (OCT) signal intensities comparison. a−c) OCT signal intensities were obtained from the Au NP-BiOI MPs (a), BiOI MPs (b), and SonoVue® microbubbles (c) distributed in the agar gel phantom tubes with different concentrations. d) Comparison of OCT signal intensities between Au NP-BiOI MPs, BiOI MPs, and SonoVue® at different concentrations.

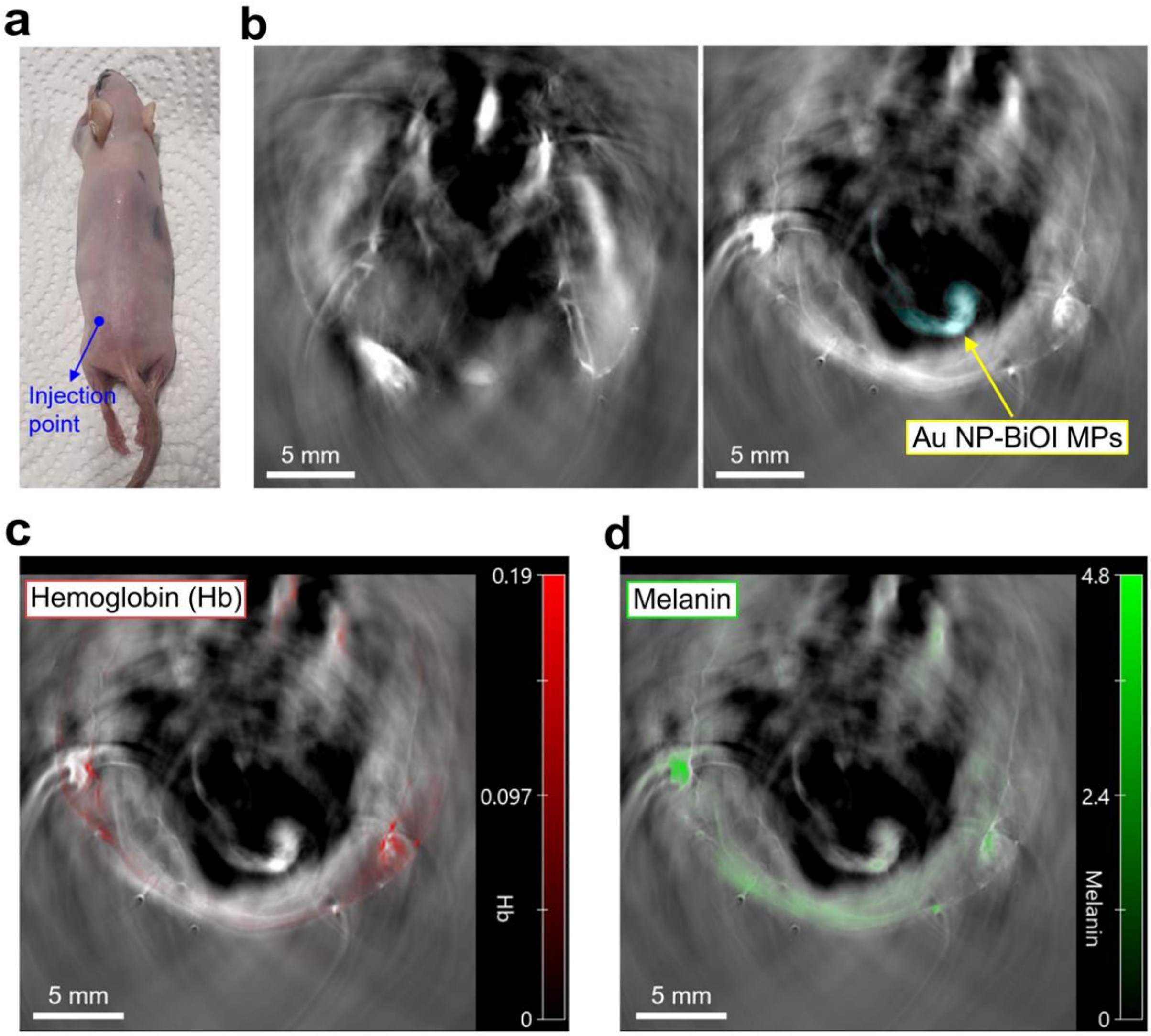

Figure S17. ***Ex vivo*** **optoacoustic tomography (OAT) imaging of the Au NP-BiOI MPs.** a) Injection of the Au NP-BiOI MPs (1.0 mg mL^{-1} in water) into the peritoneal cavity of an *ex vivo* mouse. b) OAT imaging of the mouse peritoneal cavity before (left) and after (right) injection of the Au NP-BiOI MPs. The OAT images were obtained at the wavelength of 800 nm. c,d) OAT imaging of haemoglobin (Hb) (c) and melanin (d) obtained at the same position of (b) at the wavelength of 800 nm.

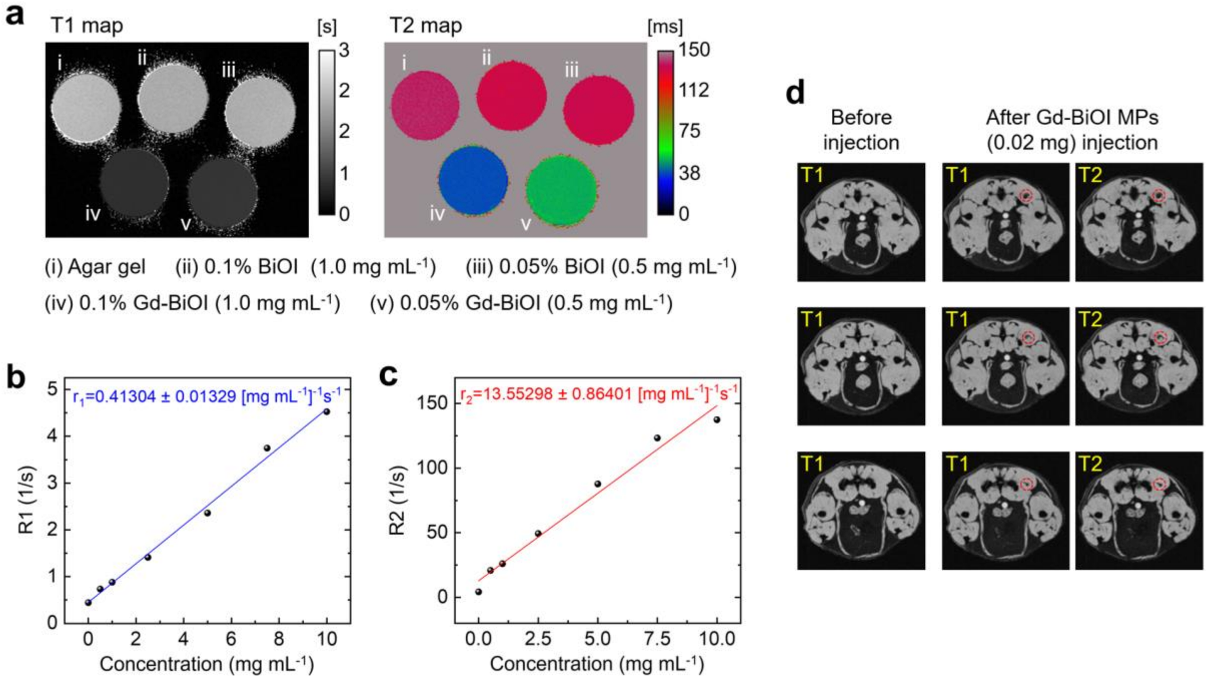

Figure S18. Magnetic resonance imaging (MRI) phantom test of BiOI MPs and Gd-BiOI MPs dispersed in agar gel with different concentrations. a) MRI scot images of five different phantom samples at T1 and T2 maps. b,c) Plot of r_1 relaxation rate (b) and r_2 relaxation rate (c) against Gd-BiOI MPs concentration of the MRI phantoms. The Gd-BiOI MPs have a less pronounced effect on the T1 (positive) contrast, decreasing slightly with increasing concentration, but a significant T2 (negative) contrast, resulting in r_2/r_1 value of 32.8. d) T1- and T2-weighted imaging of *ex vivo* mouse peritoneal cavity before and after injection of 0.02 mL of Gd-BiOI MPs (concentration of 1.0 mg mL^{-1}). The red circle is the position of injection.

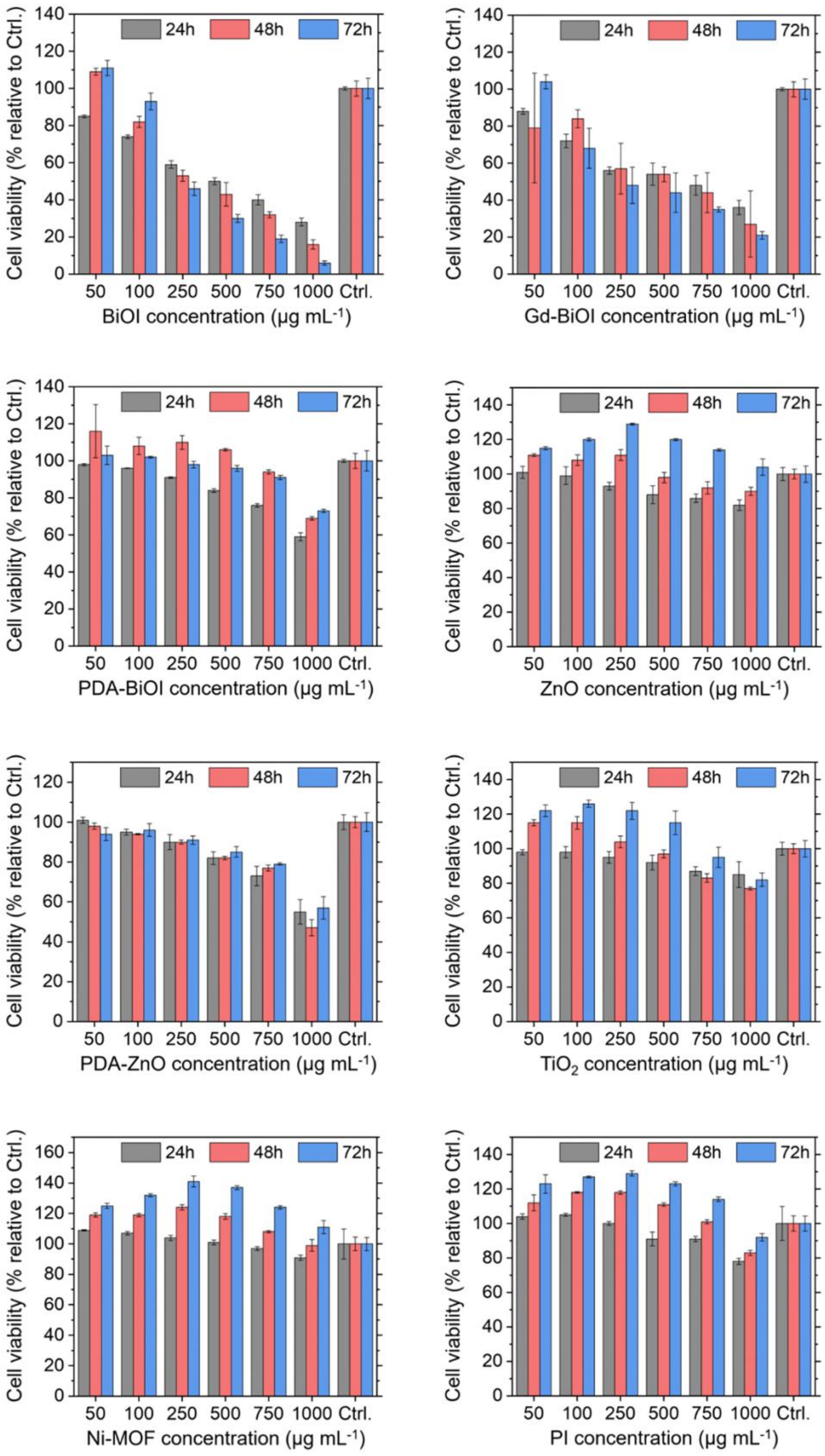

Figure S19. Cytotoxicity of various HNS-MPs. The first viability test of fibroblasts cells exposed to various concentrations (50−1000 μg mL^{-1}) of the different HNS-MPs for 24, 48, and 72 hours.

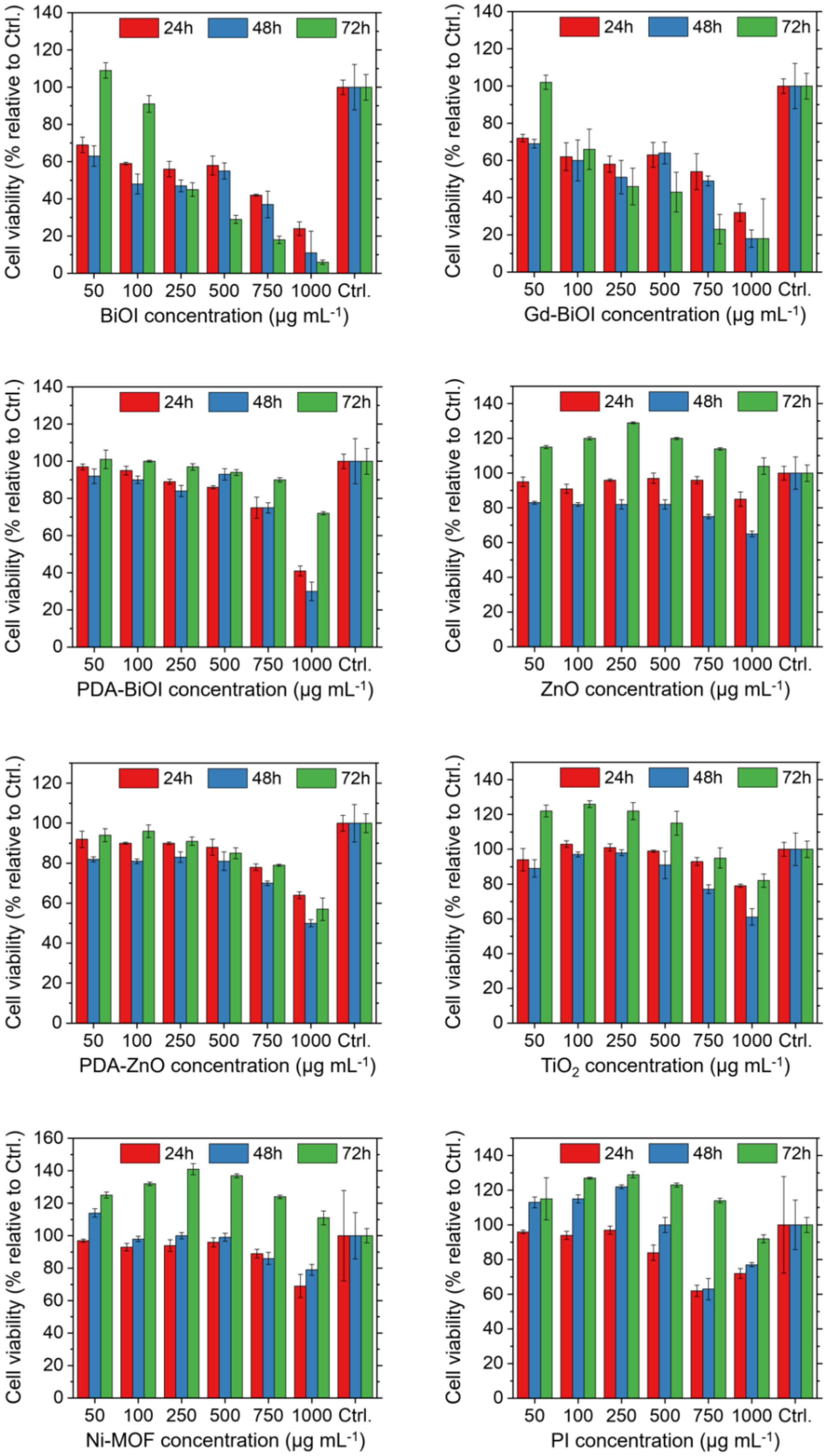

Figure S20. Cytotoxicity of various HNS-MPs. The second viability test of fibroblasts cells exposed to various concentrations (50−1000 μg mL^{-1}) of the different HNS-MPs for 24, 48, and 72 hours.

Hemocompatibility of HNS-MPs (Figures S21 and S22)

Hemocompatibility test of materials was separated into hematotoxicity and thrombogenicity studies. For hematotoxicity studies, while the blood smears were stained with hematoxylin and eosin for cell morphology investigation, luciferase assay was used for platelet activation and aggregation measurements. In this way, possible thrombogenesis and hemolysis pathways for biomaterials were investigated.

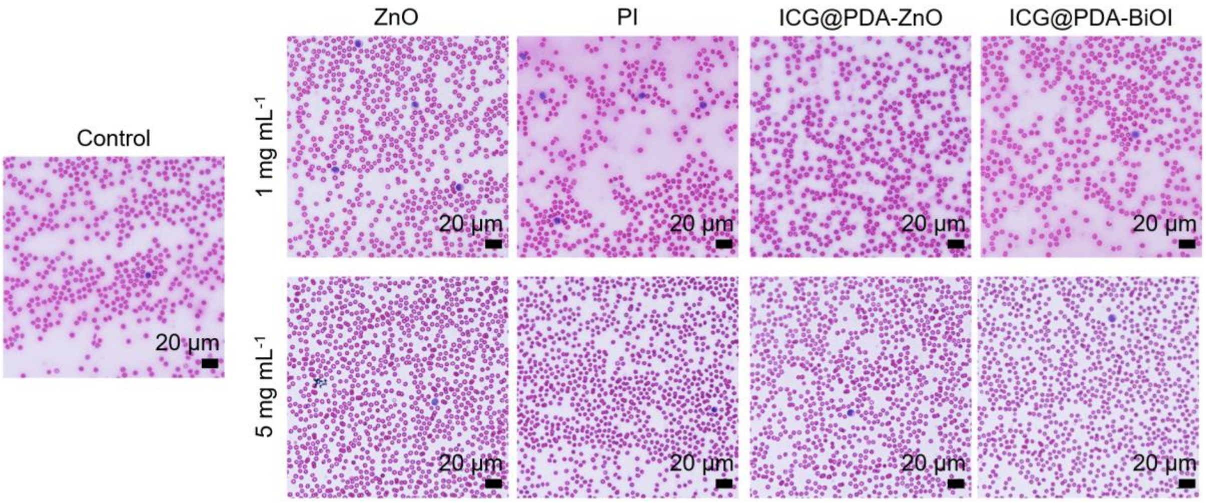

Figure S21. Hematotoxicity test of various HNS-MPs. The researchers did not observe any morphological changes in leukocytes and erythrocytes in all experimental groups after a short-time (15 minutes) agitated blood incubation period. This result indicates that the HNS-MPs did not cause any hemolytic activity or cellular damage to leukocytes and erythrocytes at both concentrations (1 and 5 mg mL^{-1}).

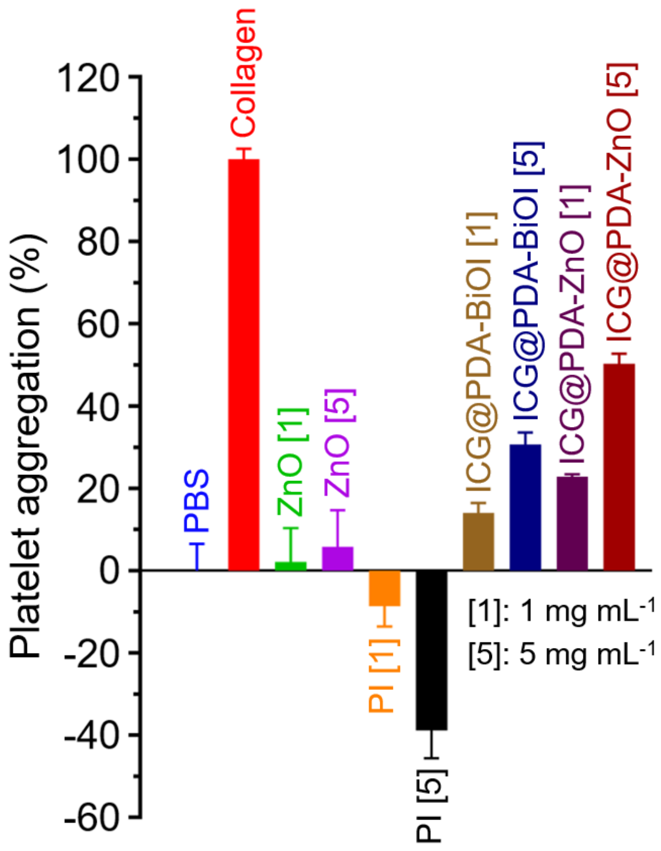

Figure S22. Thrombogenicity test of various HNS-MPs. While the platelet activation and aggregation were investigated in each experimental group, no significant thrombogenicity was observed in ZnO MPs in both concentrations (1 and 5 mg mL^{-1}). Due to the luminescent interference of the PI MPs, coagulation could not be measured in this experimental group. On the other hand, both ICG@PDA-ZnO and ICG@PDA-BiOI MPs induced mild platelet aggregation, ~20% adenosine triphosphate (ATP) release at 1.0 mg mL^{-1} concentration, that increased with concentration for both experimental groups.

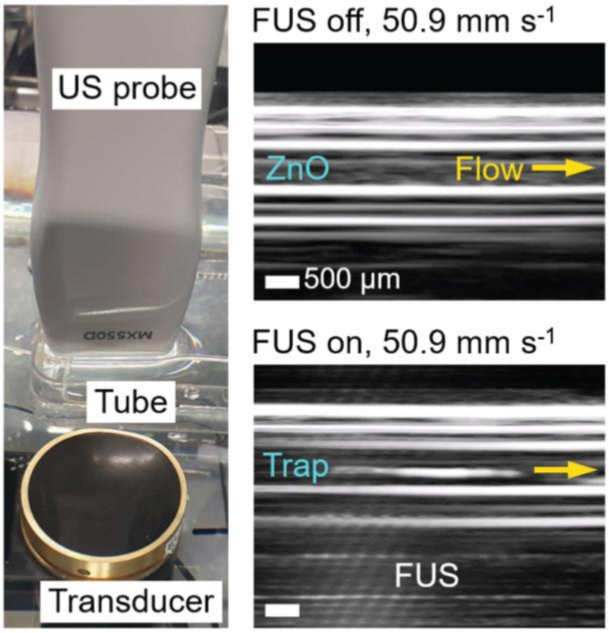

Figure S23. Real-time acoustic trapping and US imaging of ZnO MPs under flow. Left: photograph of the ultrasound imaging (US) setup. Right: US imaging of the ZnO MPs flow and their trapping under water flow.

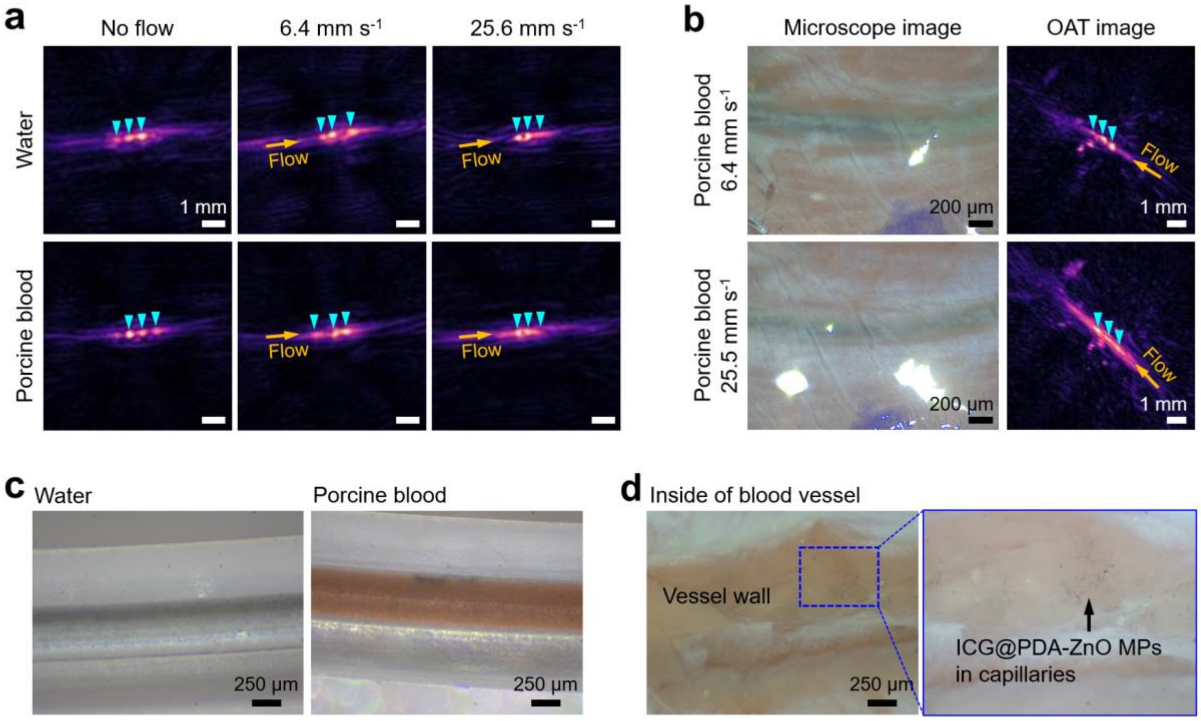

Figure S24. Real-time triple foci acoustic trapping and OAT imaging of ICG@PDA-ZnO MPs within a tube and an *ex vivo* blood vessel under the flow of water and porcine blood. a,b) OAT imaging of the triple traps of ICG@PDA-ZnO MPs within 500 µm-diameter tubes (a) and *ex vivo* blood vessels (b) under various flow velocities. c,d) Microscopy images of the walls of tubes and *ex vivo* blood vessels after releasing the traps and flushing the fluids.

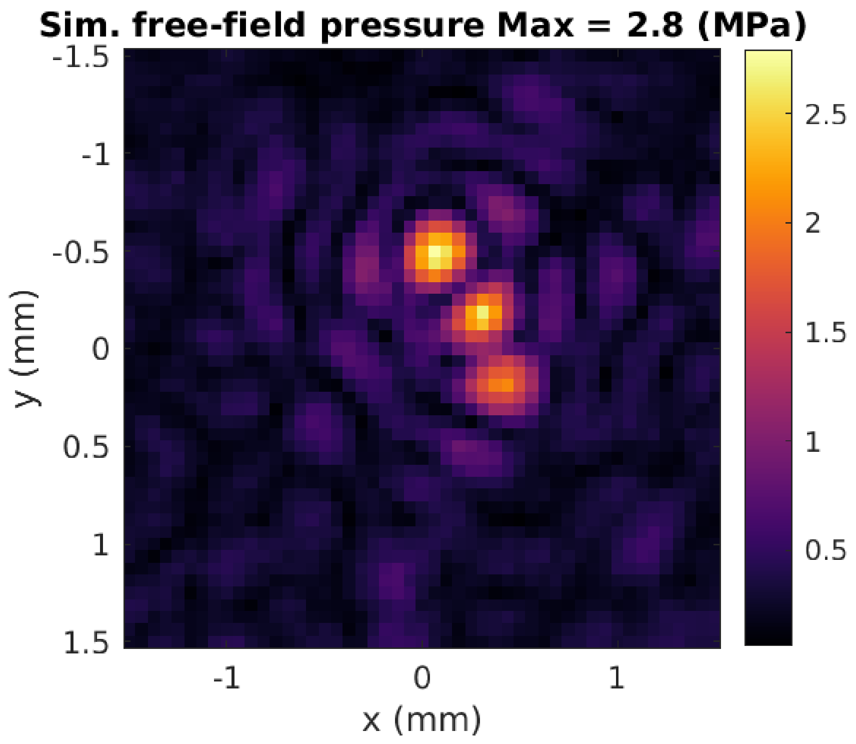

Figure S25. Simulated pressure field applied by the customized POUS system. The applied pressure by 3 foci generated by the POUS system is simulated and depicted by a customized MATLAB code. The representation is given in two-dimension (2D).

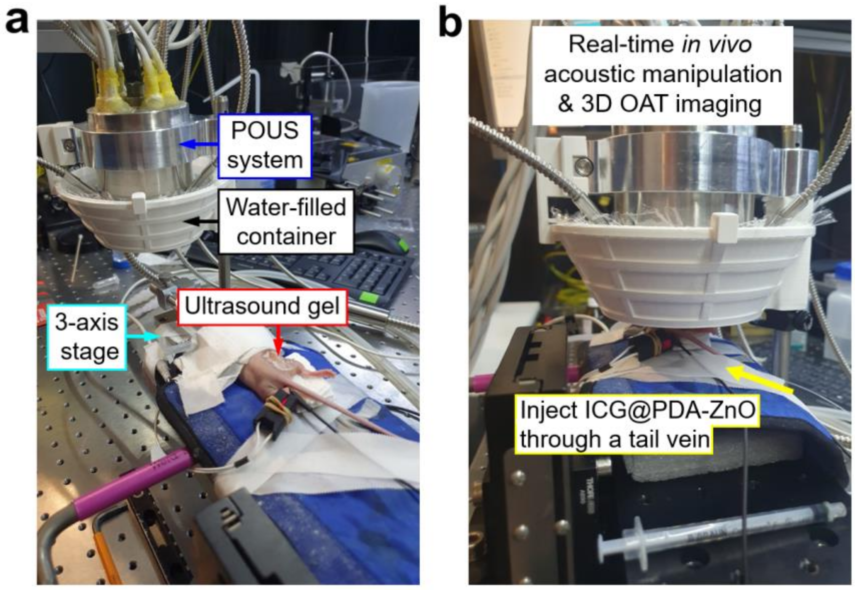

Figure S26. ***In vivo* experimental setup with POUS system.** a,b) Photographs of the setup (a) and experiment (b) showing the real-time *in vivo* acoustic manipulation and 3D OAT imaging of ICG@PDA-ZnO MPs inside a mouse femoral vein using the POUS system.

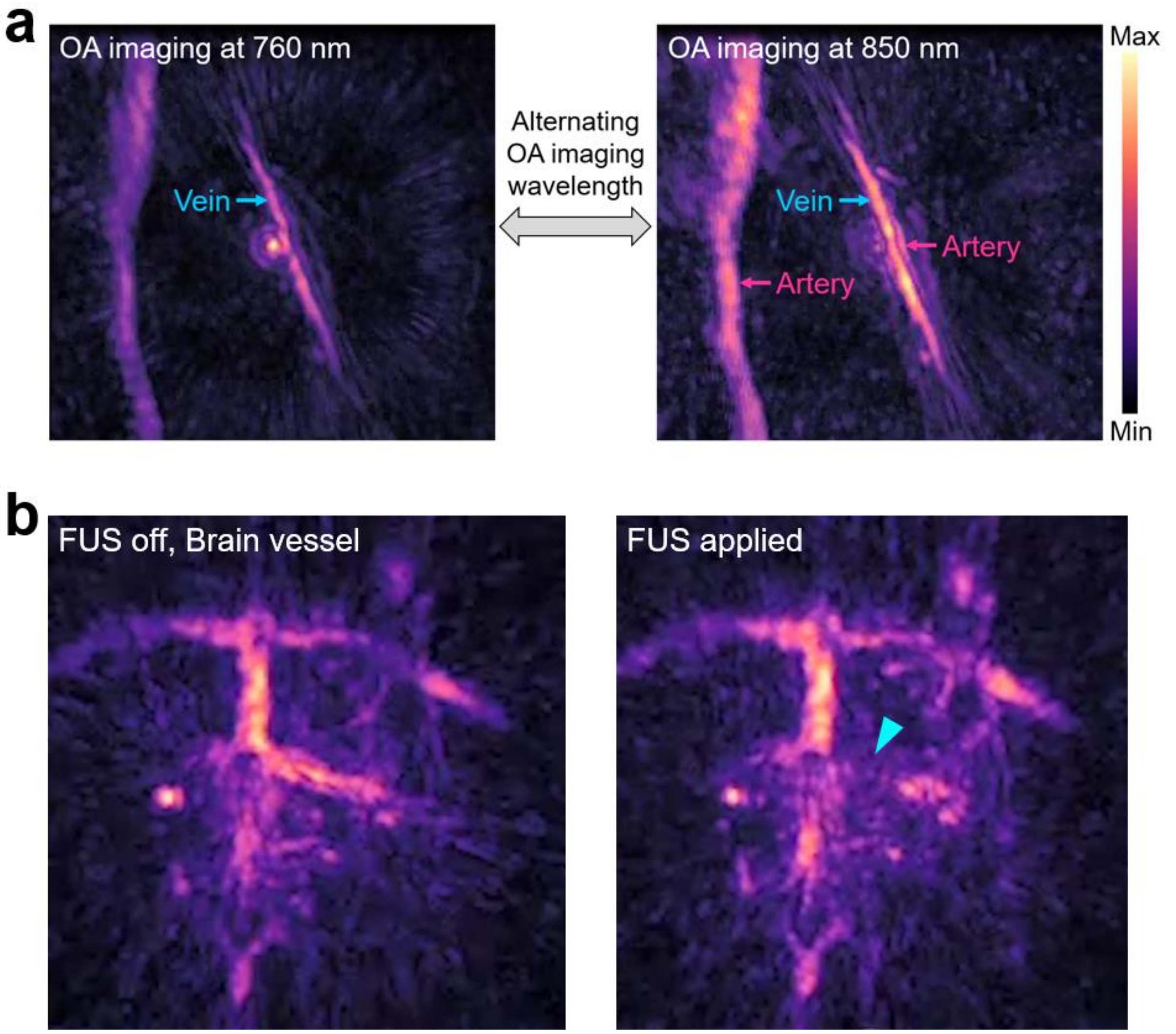

Figure S27. Distinction between arteries and veins and red blood cells displacement utilizing POUS system. a) *xy*-plane OAT imaging view of the femoral vein by alternating imaging wavelength between 760 and 850 nm. Given the close proximity between arteries to the veins, a distinct differentiation was achieved by alternating the OAT imaging wavelength between 760 and 850 nm. Deoxyhemoglobin (Hb) exhibits pronounced absorption at 760 nm, while oxyhemoglobin (HbO_2) does not. Conversely, both Hb and HbO_2 exhibit absorption at 850 nm. This OAT imaging technique, utilizing the presence of Hb and HbO_2 in veins and arteries, respectively, enabled accurate and reliable distinction between a femoral vein and surrounding arteries. b) Displacement of red blood cells in brain blood vessels of *in vivo* mice by applying FUS.

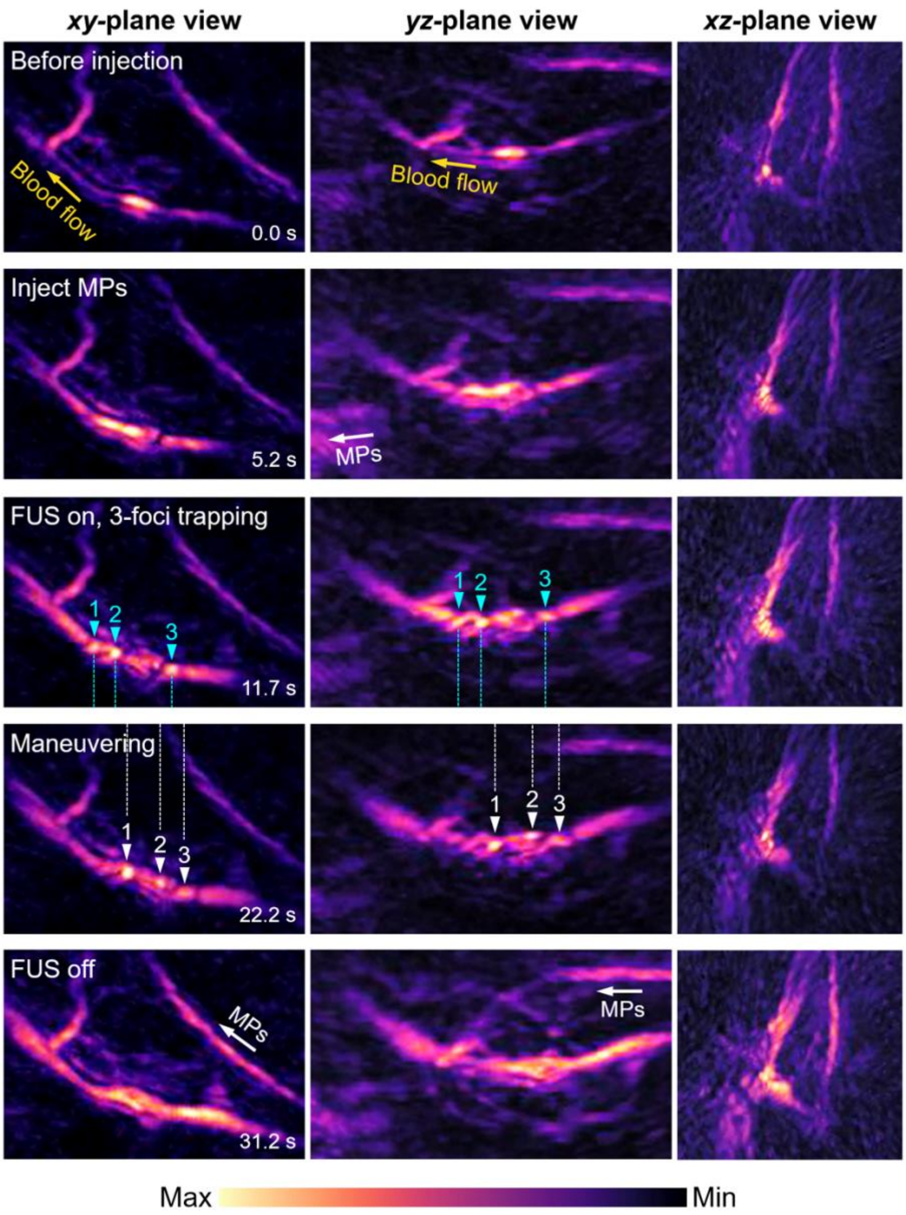

Figure S28. Real-time *in vivo* acoustic manipulation and 3D optoacoustic imaging of triple MPs traps utilizing POUS system. *xy*-, *yz*-, and *xz*-plane OAT imaging view showing the injection of ICG@PDA-ZnO MPs through the tail vein, formation of triple MP traps within the femoral vein, positional maneuvering, and dissolution of the traps.

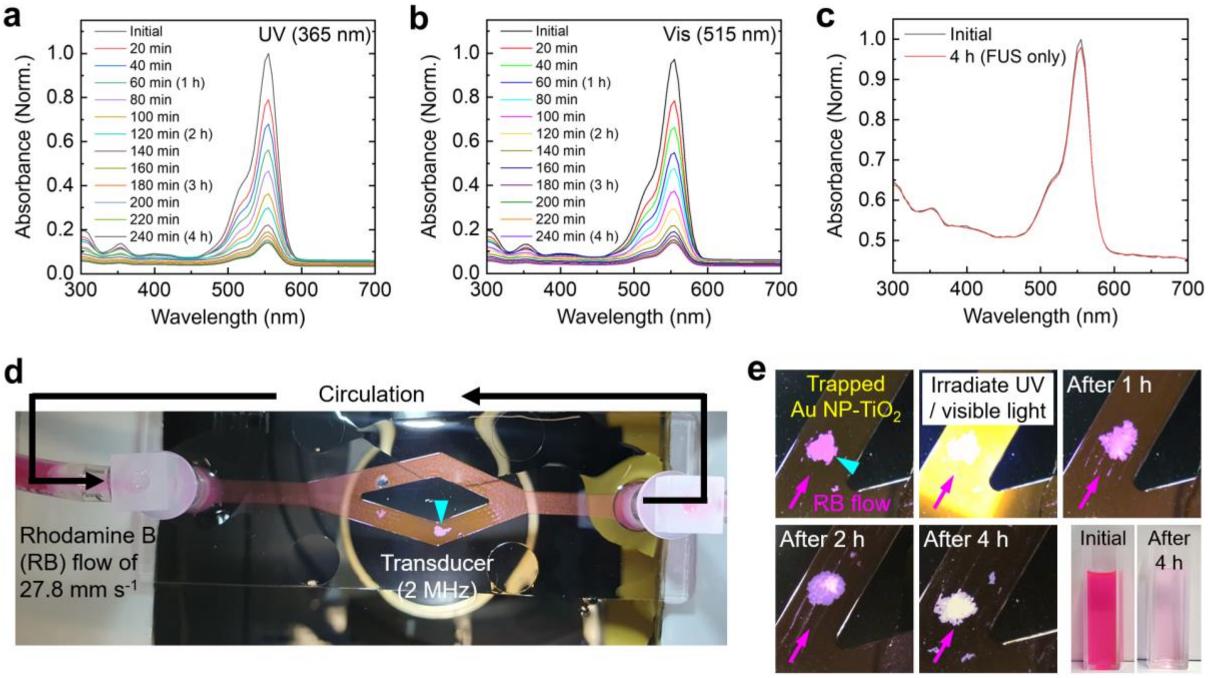

Figure S29. Photocatalytic degradation of Rhodamine B (RB) dye using Au NP-TiO_2 MPs trapped inside a microfluidic channel. a,b) Time-dependant change of absorption spectra of Rhodamine B (RB) aqueous solution (initial concentration of 0.2 µM) with the trapped Au NP-TiO_2 MPs under ultraviolet (UV) (a) and visible (b) light irradiation. The 1 mL of RB solution was taken during the experiment every 20 min and its absorption spectrum was measured by UV-Vis spectroscopy. c) Absorption spectra of the initial RB solution and after four hours only applying FUS. d) Photograph of the experimental setup for photocatalytic RB dye degradation using Au NP-TiO_2 MPs trapped inside a microfluidic channel under UV or visible irradiation. e) Photographs showing the changes in the RB solution near the trapped Au NP-TiO_2 MPs over four hors. The last photograph shows the RB solution before experiment and after four hours of experiment.

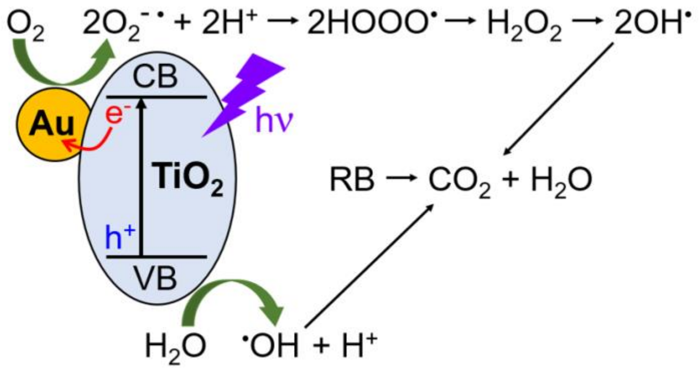

TiO_2 + hν (UV or Vis) → TiO_2^* ($e_{CB}^- + h_{VB}^+$) [1]

$H_2O + h_{VB}^+ \rightarrow {}^\bullet OH + H^+$ [2]

$O_2 + e_{CB}^- \rightarrow O_2^{-\bullet}$ [3]

RB + ($^\bullet OH$, $O_2^{-\bullet}$, or h_{VB}^+) → photodegraded intermediates + H_2O + CO_2 [4]

TiO_2^*: excited state of TiO_2

e_{CB}^-: photoexcited electron in the conduction band (CB)

h_{VB}^+: photogenerated hole in the valence band (VB)

$^\bullet OH$: hydroxyl radical

$O_2^{-\bullet}$: superoxide

Figure S30. Mechanism of Rhodamine B (RB) dye photocatalytic degradation by Au NP-TiO_2 MPs. The decomposition of RB can be described by the above mechanism. When photons (hν) are absorbed by TiO_2, electron (e_{CB}^-) and hole (h_{VB}^+) pairs can be generated inside TiO_2^* [1]. After electron-hole pairs generation, a Schottky junction at the interface of TiO_2 and Au NPs facilitates the migration of photoexcited e_{CB}^- from the conduction band in TiO_2 to Au NPs, preventing their recombination with the generated h_{VB}^+ in the valence band. The h_{VB}^+ at the valence band of TiO_2, and e_{CB}^- at the conduction band of TiO_2 and Au NPs undergo catalytic reaction with surrounding H_2O and O_2, generating hydroxy radical ($^\bullet OH$) and superoxide ($O_2^{-\bullet}$) species ([2] and [3]). $^\bullet OH$ and $O_2^{-\bullet}$ are strong oxidants and contribute to the photocatalytic decomposition of RB with h_{VB}^+ [4].

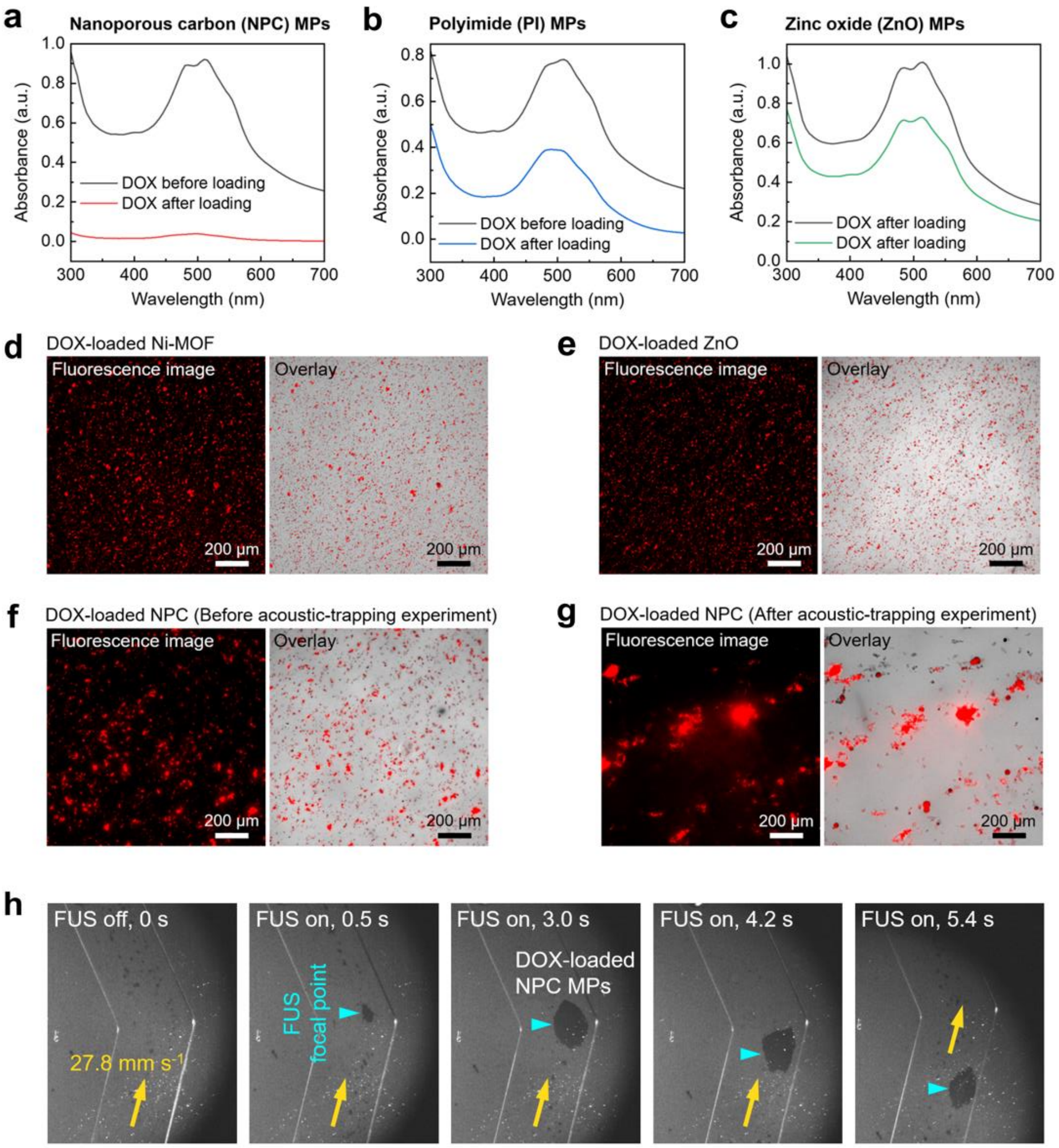

Figure S31. Loading of Doxorubicin (DOX) into the various HNS-MPs. a–c) For DOX loading efficiency, UV-Vis spectra of DOX in dPBS (0.5 mg mL^{-1}) before agitation with NPC (a), PI (b), and ZnO (c) MPs (Each MP has concentration of 2 mg mL^{-1}.) were obtained first (The curve of "DOX before loading"). Subsequently, following 24 hours stirring of DOX solutions with MPs to facilitate DOX coating or absorption, the suspension underwent centrifugation, and a UV-Vis spectrum of the supernatant was obtained (The curve of "DOX after loading"). d–g) Fluorescent images of the various DOX-loaded MPs overlaid with their bare microscopy images. In order to obtain fluorescent images, the DOX-loaded MPs were illuminated with 475 nm-wavelength light using a fluorescent microscope. (f) and (g) shows the fluorescent images of the DOX-loaded NPC MPs before and after underwent acoustic-trapping experiments, respectively. h) Acoustic trapping and manipulation of the DOX-loaded NPC MPs in the microfluidic channels under water flow velocity of 27.8 mm s^{-1}.

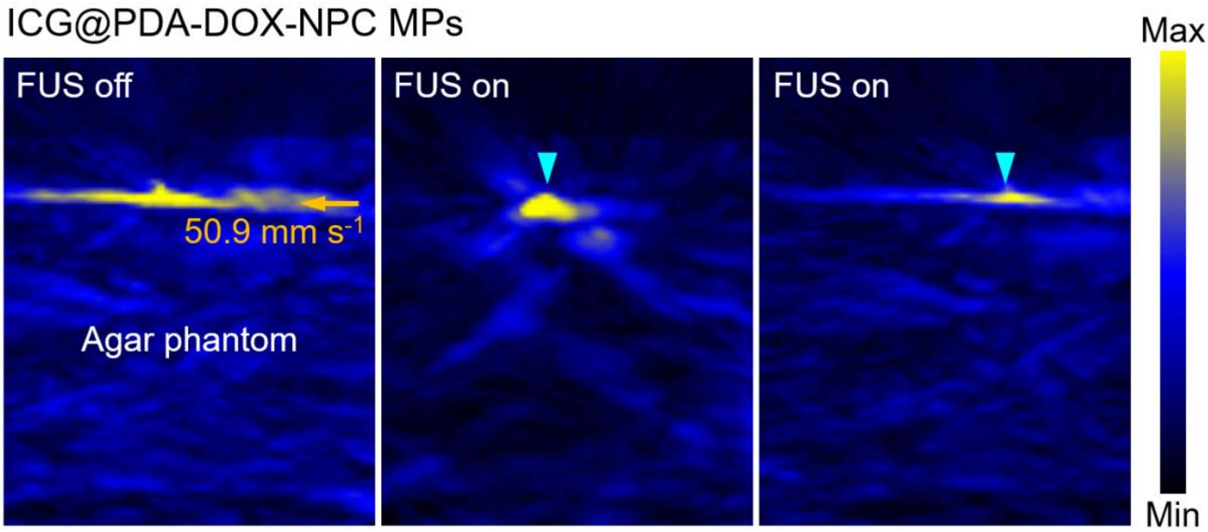

Figure S32. OAT imaging of trapping and manipulation of the ICG@PDA NPs-coated, DOX-loaded NPC MPs (ICG@PDA-DOX-NPC MPs) under an established flow of 50.9 mm s^{-1} velocity. The ICG@PDA-DOX-NPC MPs were trapped and manipulated within the tube inserted inside an agar phantom. For FUS-driven trapping, a 2 MHz and 0.42 MPa single-beam transducer was used. The presented images are top-view images.

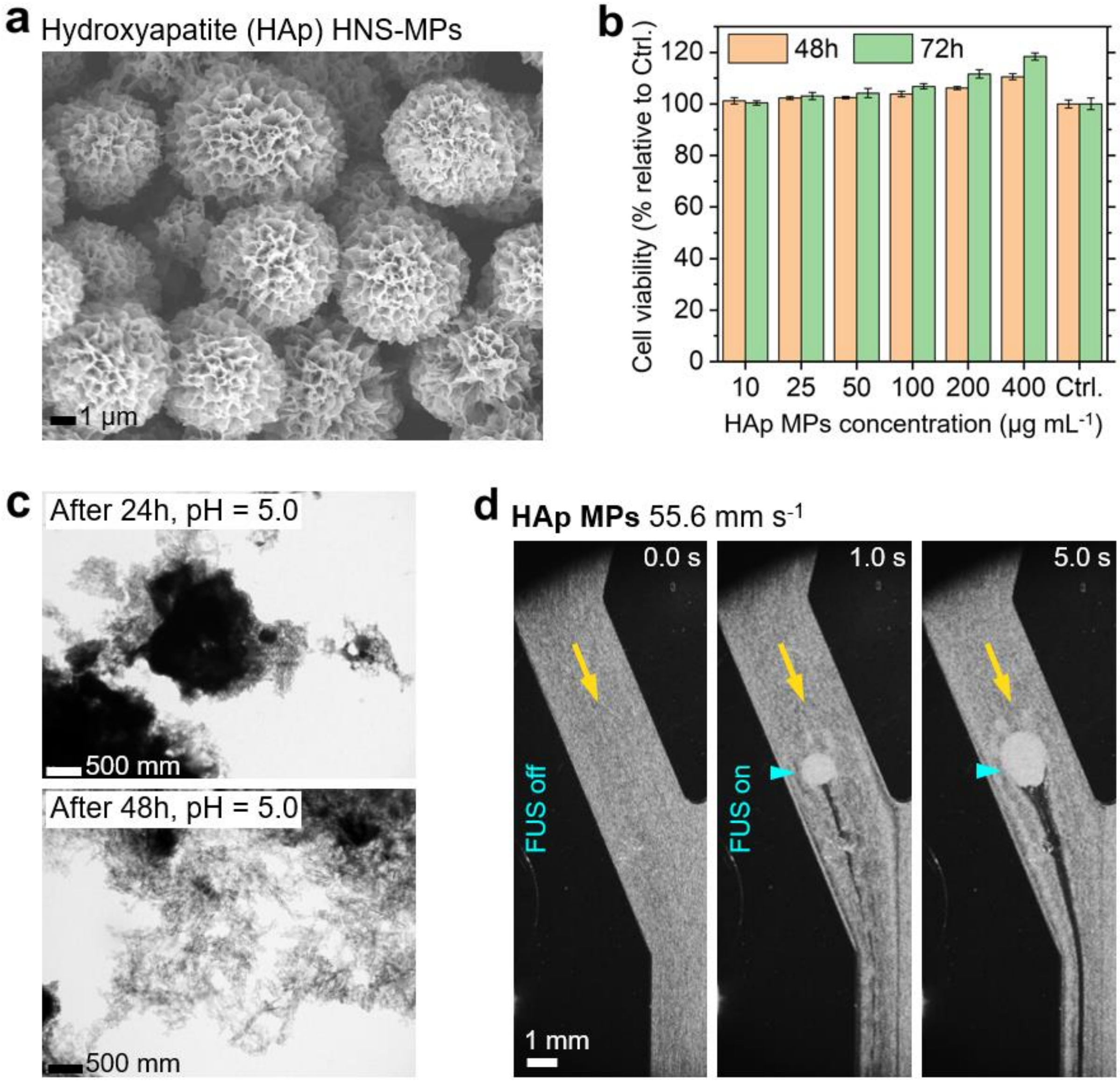

Figure S33. Flower-like hydroxyapatite ($Ca_5(PO_4)_3(OH)$, HAp) HNS-MPs with high cell viability, pH-degradability, and acoustic manipulation capability. a) SEM image of the HAp HNS-MPs. b) Viability test of fibroblasts cells exposed to various concentrations (10‒400 μg mL^{-1}) of the HAp MPs for 48 and 72 hours. c) TEM images showing the degradation of HAp MPs after 24 and 48 hours in pH = 5.0 environments. d) Time-dependent photographs showing the formation of a HAp MPs trap under an established water flow with the velocity of 55.6 mm s^{-1}.

Supporting Movies

Movie S1. Acoustic trapping and manipulation in fluid flow: Solid MPs VS Various HNS-MPs

Movie S2. Side-view of trapped ZnO HNS-MPs in 500 μm-diameter tubes under static/dynamic flow conditions

Movie S3. Acoustic trapping of HNS-MPs in larger tubes under fluid flow

Movie S4. Acoustic trapping and manipulation of PI HNS-MPs in microfluidic channels

Movie S5. Acoustic trapping and manipulation of ZnO HNS-MPs in microfluidic channels

Movie S6. Ultrasound imaging of Gd-BiOI MPs injected into *ex vivo* tumour vessels and ZnO MPs trapping under fluid flow

Movie S7. Real-time three-foci acoustic trapping and optoacoustic imaging of ICG@PDA-ZnO MPs inside tubes using POUS system

Movie S8. Real-time optoacoustic imaging of *in vivo* mouse femoral vein upon FUS activation before and after ICG@PDA-ZnO MPs injection

Movie S9. Real-time acoustic manipulation & 3D optoacoustic imaging of ICG@PDA-ZnO MPs inside *in vivo* mouse femoral vein using POUS system
